# Conserved reduction of m^6^A RNA modifications during aging and neurodegeneration is linked to changes in synaptic transcripts

**DOI:** 10.1073/pnas.2204933120

**Published:** 2023-02-22

**Authors:** Ricardo Castro-Hernández, Tea Berulava, Maria Metelova, Robert Epple, Tonatiuh Peña Centeno, Julia Richter, Lalit Kaurani, Ranjit Pradhan, M. Sadman Sakib, Susanne Burkhardt, Momchil Ninov, Katherine E. Bohnsack, Markus T. Bohnsack, Ivana Delalle, Andre Fischer

**Affiliations:** ^a^Department for Epigenetics and Systems Medicine in Neurodegenerative Diseases, German Center for Neurodegenerative Diseases, 37077 Göttingen, Germany; ^b^Department of Neurobiology, Max-Planck Institute for Biophysical Chemistry, 37077 Göttingen, Germany; ^c^Department of Molecular Biology, University Medical Center, 37077 Göttingen, Germany; ^d^Multiscale Bioimaging Cluster of Excellence, University of Göttingen, 37077 Göttingen, Germany; ^e^Department of Pathology, Lifespan Academic Medical Center, Brown University, 02912 Providence, Rhode Island; ^f^Department of Psychiatry and Psychotherapy, University Medical Center, 37077 Göttingen, Germany

**Keywords:** epi-transcriptomics, epigenetics, neuro-epigenetics, Alzheimer’s disease, RNA-methylation

## Abstract

N^6^-methyladenosine (m^6^A) regulates mRNA metabolism. While it has been implicated in the development of the mammalian brain and in cognition, the role of m^6^A in synaptic plasticity, especially during cognitive decline, is not fully understood. In this study, we employed methylated RNA immunoprecipitation sequencing to obtain the m^6^A epitranscriptome of the hippocampal subregions CA1, CA3, and the dentate gyrus and the anterior cingulate cortex (ACC) in young and aged mice. We observed a decrease in m^6^A levels in aged animals. Comparative analysis of cingulate cortex (CC) brain tissue from cognitively intact human subjects and Alzheimer’s disease (AD) patients showed decreased m^6^A RNA methylation in AD patients. m^6^A changes common to brains of aged mice and AD patients were found in transcripts linked to synaptic function including calcium/calmodulin-dependent protein kinase 2 (*CAMKII*) and AMPA-selective glutamate receptor 1 (*Glua1*). We used proximity ligation assays to show that reduced m^6^A levels result in decreased synaptic protein synthesis as exemplified by *CAMKII* and GLUA1. Moreover, reduced m^6^A levels impaired synaptic function. Our results suggest that m^6^A RNA methylation controls synaptic protein synthesis and may play a role in cognitive decline associated with aging and AD.

## Significance

The precise role of m^6^A RNA methylation in the adult brain is not well understood. In our study, we describe the genome-wide m^6^A epitranscriptome in the healthy and diseased brains of mice and humans. Our data demonstrate that a substantial amount of m^6^A transcripts are conserved. These transcripts are linked to the regulation of synaptic processes and are localized to synapses. We detected decreased m^6^A RNA methylation in brain tissue from an AD mice model and in human brain tissue from the cingulate gyrus in individuals with Alzheimer’s disease. At the mechanistic level, we provide evidence that supports that reduced m^6^A-modified transcripts are linked to impaired synaptic protein synthesis.

Decades after it was first described, the posttranscriptional modification of mRNA has recently become an area of intense research interest in multiple fields (1). N^6^-methyladenosine (m^6^A) is the most abundant internal RNA modification and has been studied in the context of multiple cellular functions (2–4). The deposition of m^6^A modifications on targeted mRNAs is mediated by the activity of a m^6^A methylation complex composed of methyltransferases (METTL) METTL3 and METTL14 with the adaptor protein WTAP (5–7). m^6^A sites are commonly located within the DRACH consensus sequence (where D = A, T, or G, R = A or G, and H = A, T, or C) and are found across the entire transcript but often concentrate close to the stop codon and in the 3′UTR (2, 3, 8). m^6^A modifications are removed by demethylases such as fat mass and obesity-associated protein (FTO) and alpha-ketoglutarate–dependent dioxygenase AlkB homologue 5 (ALKBH5), making the regulation of m^6^A levels a complex and dynamic process (9, 10). Several reader proteins recognize m^6^A-labeled transcripts affecting a broad array of biological processes associated with mRNA metabolism, including nuclear export, transport, degradation, and translation (7, 11–14). There is also emerging evidence that such epitranscriptomic mechanisms play a role in the onset and progression of various diseases including malignant neoplasms (15). m^6^A RNA methylation has been linked to synapse function, memory consolidation, fear extinction, and recovery from brain injury (16–23). More recently, the expression levels of the methylation machinery and m^6^A levels have been investigated in the context of neurodegenerative diseases, particularly in Alzheimer’s disease (AD) (24–27). While changes in m^6^A levels have been observed in AD, the magnitude of these changes and their consequences for disease progression are not fully understood (28–30). Thus, more research on the epitranscriptome in the healthy and diseased brains is needed.

In this study, we analyze the m^6^A epitranscriptome in adult mouse and human brains. We found a conservation of methylation sites in transcripts that are linked to synapse function. Differential methylation analyses of brain regions of aged mice and human AD patients revealed a substantial reduction of m^6^A RNA methylated transcripts specifically within transcripts involved in synapse functioning such as *CAMKII* or *Glua1*. In line with these data, we show that reducing m^6^A levels impairs synaptic plasticity and results in reduced synaptic translation of these *CAMKII* and *Glua1* transcripts into the corresponding protein. These data suggest that loss of m^6^A modifications on transcripts associated with synaptic function and plasticity could be an early event in cognitive diseases.

## Results

### m^6^A Landscape in the Adult Mouse Brain.

**Fig. 1.**
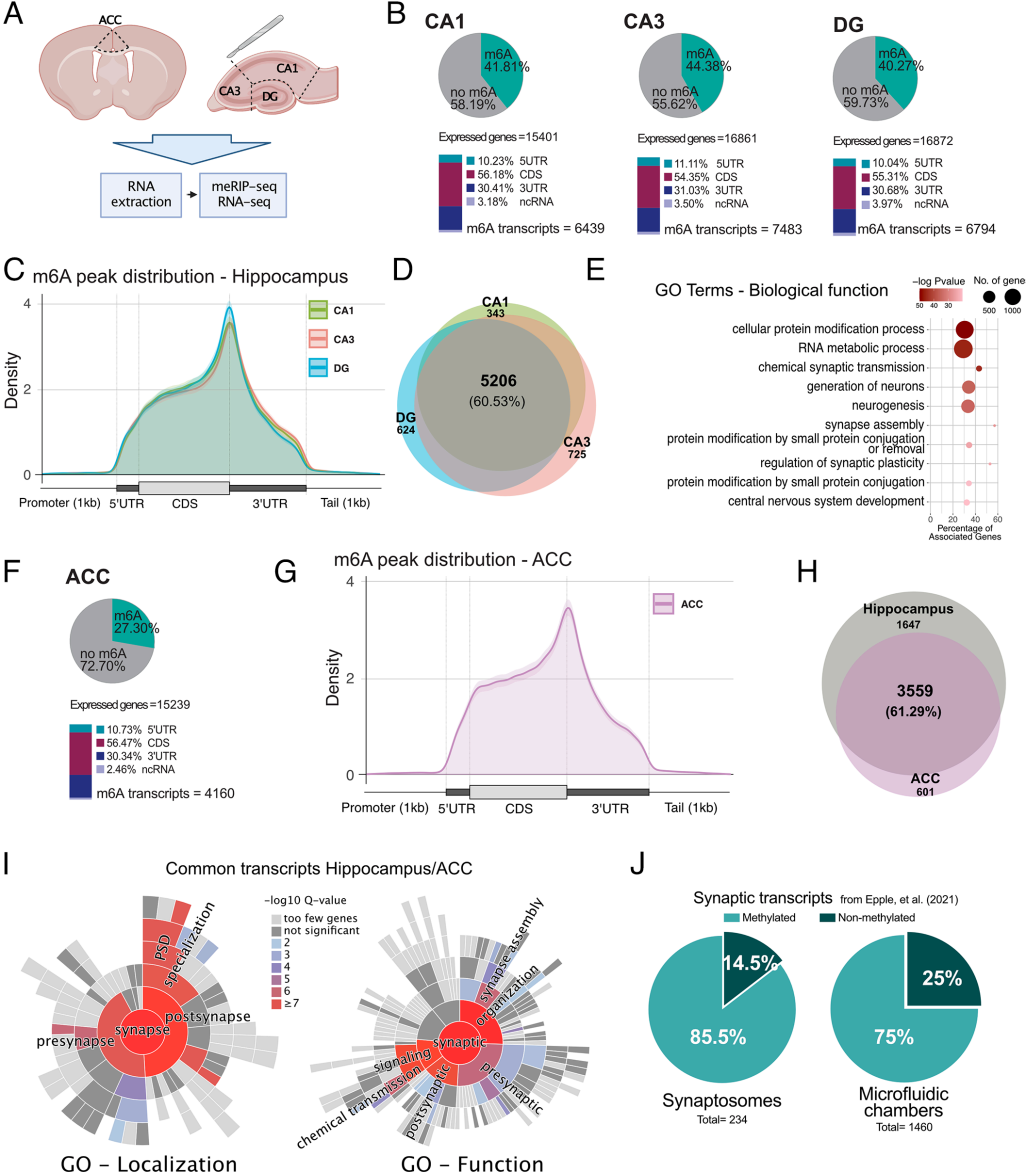
The m^6^A epitranscriptome in the adult mouse brain. (*A*) Experimental scheme. (*B*) *Upper*: Pie chart showing the percentage of m^6^A methylated transcripts in the hippocampal CA1, CA3, and DG regions when calculated against the corresponding input. *Lower*: m^6^A peak location is shown for the annotated transcript regions. The percentages are calculated from the total number of m^6^A peaks. (*C*) Guitar plot showing the distribution of m^6^A peaks along mRNAs in the hippocampal subregions. (*D*) Venn diagram comparing m^6^A methylated transcripts across hippocampal subregions. (*E*) GO term (biological function) analysis of the m^6^A methylated transcripts commonly detected in all hippocampal regions.*(G)* Guitar plot showing the distribution of m^6^A peaks along mRNAs in the ACC. (*F*) *Upper*: Distribution of m^6^A RNA methylation across the transcriptome in the ACC. *Lower*: m^6^A peak location is shown for the annotated transcript regions. The percentages are calculated from the total number of m^6^A peaks. (*H*) Venn diagram comparing m^6^A commonly methylated transcripts in the hippocampus and ACC. (*I*) Sunburst plots showing the enrichment of synapse-specific GO terms within commonly m^6^A methylated transcripts in the hippocampus and ACC. (*J*) Pie chart showing the percentage of m^6^A methylated transcripts within RNA-seq datasets obtained from hippocampal synaptosomes or synapses isolated from microfluidic chambers. ACC - anterior cingulate cortex, DG - dentate gyrus, 5UTR - 5′ untranslated region, 3UTR - 3′ untranslated region, CDS - coding sequence, ncRNA - noncoding RNA.

We started our analysis by characterizing the landscape of m^6^A RNA modifications in the healthy adult mouse brain. The brains of ten (C57BL/6J) 3-mo-old (young) wild-type (WT) mice were extracted and dissected to obtain hippocampal subregions CA1, CA3, and dentate gyrus (DG) and the anterior cingulate cortex (ACC), which have been implicated with learning and memory processes and cognitive disease (Fig. 1*A*) (31). Methylated RNA immunoprecipitation sequencing (meRIP-seq) was performed to determine the subregion-specific epitranscriptomic landscape in young adult mice.

The analysis of methylated regions revealed a large number of m^6^A peaks in the hippocampal brain subregions, with 18,270 peaks detected in the CA1, 20,415 in the CA3, and 16,686 in the DG (*SI Appendix*, Fig. S1*A*). These data are in agreement with previous findings from genome-wide analysis of m^6^A levels in cerebellar and cortical mouse tissues (16, 20, 22). When we compared the transcripts carrying m^6^A modifications to the entire transcriptome, we observed that 40.27% of the expressed genes in the DG were modified by m^6^A RNA methylation, while in the CA1 and CA3 regions, 42.81% and 44.38% were detected, respectively (Fig. 1*B* and Dataset S1). On average, every methylated transcript had 2.4 to 2.7 m^6^A methylated regions per transcript depending on the hippocampal subregion (*SI Appendix*, Fig. S1*B*). Motif enrichment analyses of the detected m^6^A peaks showed a strong overrepresentation of the m^6^A DRACH consensus motif, confirming that meRIP-seq had successfully enriched for m^6^A sites (*SI Appendix*, Fig. S1*C*). The m^6^A modifications were detected across the gene bodies and enriched in the vicinity of the stop codon and 3′UTR (Fig. 1 *B* and *C*), which is in agreement with previous data (2). About 60% (5,206 transcripts) of the transcripts with m^6^A modifications could be detected in all hippocampal subregions (Fig. 1*D* and Dataset S2). Gene ontology (GO) term analysis revealed that the m^6^A-modified transcripts detected in all hippocampal subregions showed a significant enrichment for genes associated with synaptic transmission, neurogenesis, synapse assembly, and RNA metabolism (Fig. 1*E* and Dataset S3). For example, more than 332 transcripts were linked to chemical synaptic transmission including genes such as **CAMKII*a,* or *Glua1 (also known as Gria1)* that were methylated in all hippocampal subregions. We also detected transcripts that were exclusively m^6^A modified in only one of the hippocampal subregions. In detail, 725 transcripts were specifically methylated in the CA3 region, 624 in the DG region, and 343 in the CA1 region (Fig. 1*D* and Dataset S4). GO term analysis revealed that CA1-specific transcripts are enriched for only two processes, namely negative regulation of protein complex assembly and regulation of sodium ion transport (*SI Appendix*, Fig. S1*D* and Dataset S5). CA3-specific transcripts with m^6^A modifications were linked to biological processes such as nuclear export, protein localization to the cellular periphery, or regulation of autophagy (*SI Appendix*, Fig. S1*E* and Dataset S6). Analysis of DG-specific transcripts revealed a number of interesting GO terms of which noncoding (nc) RNA processing was the most significant (*SI Appendix*, Fig. S1*F* and Dataset S7).

To further understand how the epitranscriptomic landscape varies across brain subregions, we performed meRIP-seq analysis from ACC tissue obtained from the same young mice. In the ACC, 11,816 m^6^A peaks were detected corresponding to 4,160 consistently methylated transcripts (2.83 peaks per transcript), which represented 27.3% of the expressed genes (Fig. 1*F* and *SI Appendix*, Fig. S1 *A* and *B*).

The distribution of m^6^A modifications across transcripts detected in the ACC was similar to the pattern observed for the hippocampal tissues (Fig. 1 *F* and *G*). Considering all transcripts methylated in the hippocampus and ACC, 61.29% were methylated in both (Fig. 1*H* and Dataset S8). Methylation sites also displayed similar m^6^A levels in hippocampal brain regions, especially when comparing the CA1 and CA3 regions, while the DG was more distant. The ACC was more distant from the hippocampal regions, and all investigated brain regions substantially differed from the heart, another mainly postmitotic and excitable organ (*SI Appendix*, Fig. S1 *G* and *H*). GO term analysis revealed that transcripts commonly methylated in the hippocampus and ACC showed a strong enrichment for pathways associated with synaptic assembly, organization and signaling, and learning and memory (*SI Appendix*, Fig. S2*A* and Dataset S9). Using SynGO (32), an experimentally annotated database for synaptic location and functional GO, we observed that transcripts commonly methylated in the hippocampus and ACC are highly enriched for proteins known to be localized to synapses (Fig. 1*I*). ACC-specific transcripts also displayed a significant enrichment for synaptic proteins, and the most significant GO terms linked to the m^6^A transcripts specific to the ACC were also related to synapse function (*SI Appendix*, Fig. S2*B* and Dataset S10). While chemical synaptic transmission was the most significant GO term when transcripts common to the ACC and hippocampus were analyzed, modulation of chemical synaptic transmission and chemical synaptic transmission were the most enriched GO terms for m^6^A transcripts specific to the ACC (*SI Appendix*, Fig. S2 *A*–*C* and Datasets S9–S11). These data may suggest that some of the transcripts specifically methylated in the ACC act in similar pathways as in the hippocampus. A closer look at the transcripts provides some evidence for this interpretation. For example, synaptotagmins (syt) control synaptic vesicle endocytosis. While the *Syt2*, *Syt4*, and *Syt13* transcripts carry m^6^A modifications in the ACC and hippocampus, in the ACC, *Syt 1*, *Syt5*, and *Syt7* were additionally methylated (Dataset S8). In contrast, hippocampus-specific transcripts showed no significant synaptic enrichment (*SI Appendix*, Fig. S2*D*).

These data suggest that common m^6^A transcripts might be specifically enriched at synapses, which is in agreement with previous data (20, 23). To further explore this notion, we made use of a recently published dataset containing a high-confidence hippocampal synaptic RNAome and compared it to our hippocampal epitranscriptome data. This synaptic RNA dataset was generated from purified synaptosomes of WT mice and primary neurons grown in microfluidic chambers to isolate their synaptic compartments, making it a robust resource of synapse-localized RNAs (33) (*SI Appendix*, Fig. S2 *E* and *F*). In both datasets, we observed a strong enrichment of methylated transcripts in synapse-localized mRNAs with more than 70% of the synaptosomes and 64% of the microfluid chamber transcriptome having at least one m^6^A peak (Fig. 1*J* and Dataset S12).

These data support previous findings suggesting m^6^A RNA modification as a crucial regulatory process of synaptic function in the adult brain (20, 23). However, it has to be mentioned that our interpretation is currently based on cross-intersecting our lists of transcripts with other databases.

### m^6^A Landscape in the Adult Human Brain Reveals a Conserved Enrichment of Transcripts Linked to Synaptic Function.

**Fig. 2.**
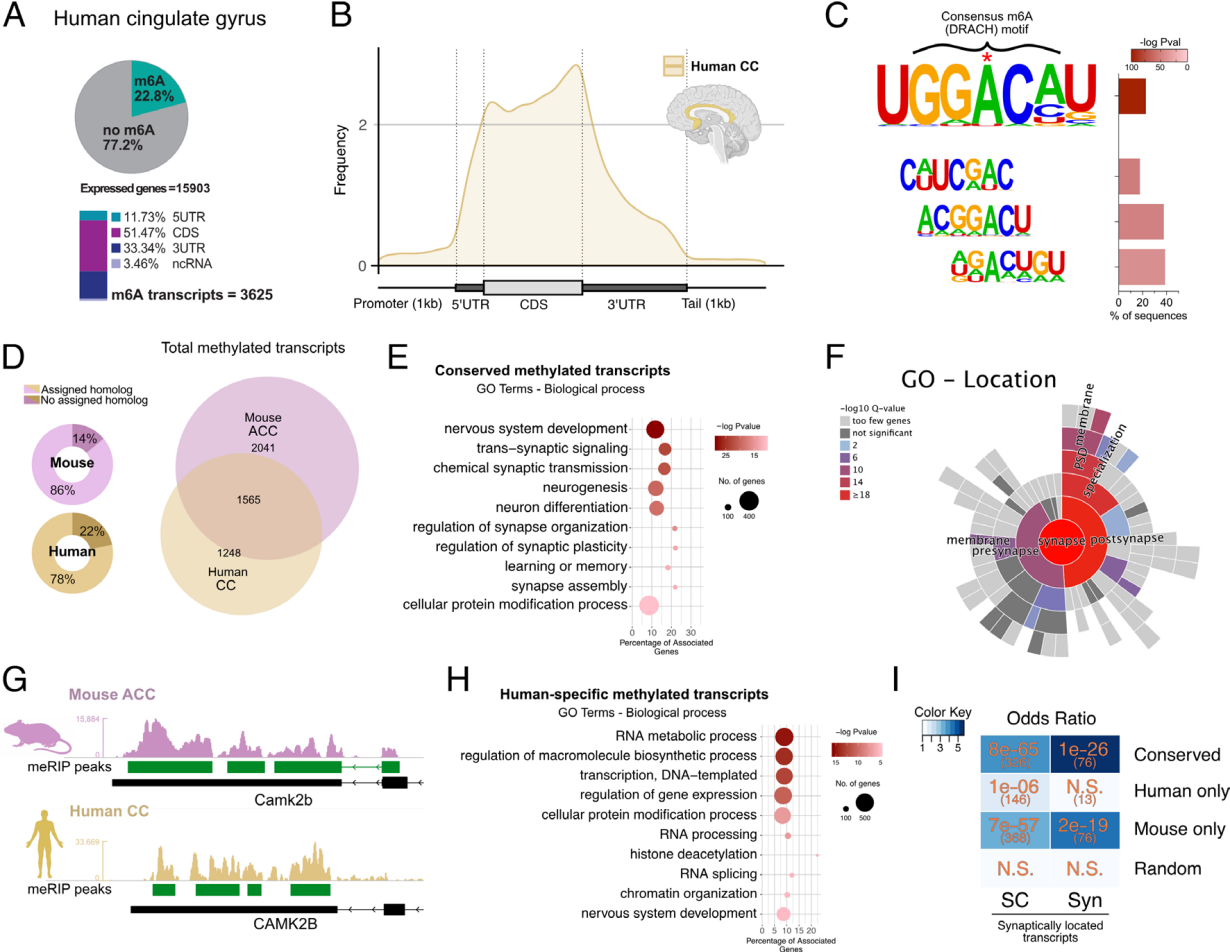
Conserved m^6^A modifications between mouse and human. (*A*) *Upper*: Pie chart showing the percentage of m^6^A methylated transcripts in the human CC calculated against the corresponding input. *Lower*: m^6^A peak location is shown for the annotated transcript regions. The percentages are calculated from the total number of m^6^A peaks. (*B*) Guitar plot showing the distribution of m^6^A modifications along mRNAs in the human CC. (*C*) Motif analysis within the m^6^A peaks identifies the m^6^A DRACH consensus motif (D = A, T, or G, R = A or G, and H = A, T, or C). (*D*) *Left*: Doughnut chart showing the mouse/human genes with known homologues in human/mouse, respectively, that were used to compare methylated transcripts across species. *Right*: Venn diagram comparing m^6^A methylated homologue transcripts in the adult mouse ACC to the human CC. (*E*) GO categories (biological process) for m^6^A methylated transcripts common to the mouse ACC and human CC. (*F*) Sunburst plot showing synapse-specific location GO term enrichment for m^6^A methylated transcripts common to the mouse ACC and human CC. (*G*) Representative coverage tracks showing conserved m^6^A modifications along the 3′ end of homologous transcripts, in this case *Camk2b* in the mouse ACC and human CC (*CAMKII*b/*CAMKII*B). Tracks show coverage values for m^6^A-RIP normalized for the corresponding inputs and library size. Scale in RPM. (*H*) GO categories (biological process) detected when m^6^A methylated transcripts specific to the human CC are analyzed. (*I*) Heat map showing the odds ratio for the association between conserved transcripts (commonly detected in mice and humans) and human- and mouse-specific transcripts in comparison to synaptic RNAs, as published in ref. 34. Color scale represents the numerical value of enrichment (odds ratio), numbers in orange correspond to the *P* value for the corresponding overlap, and numbers in parentheses refer to the number of overlapping genes. N.S. = not significant. SC = RNAs detected in the synaptic compartments of microfluidic chambers; Syn = RNAs detected in synaptosomes (34). Random corresponds to 2,000 randomly selected brain-expressed human genes. ACC - anterior cingulate cortex, CC - cingulate cortex.

Next, we decided to profile the m^6^A distribution across transcripts of the human brain employing postmortem tissue of the cingulate cortex (CC) from five nondemented individuals. We observed that 22.8% of all expressed transcripts (3,625) carried at least one m^6^A peak (Fig. 2*A*). This corresponded to 11,672 detected m^6^A peaks, with an average of 3.17 peaks per methylated transcript (*SI Appendix*, Fig. S3*A*). Similar to our observation in mice, m^6^A peaks were detected across the CDS with an enrichment in the vicinity of the stop codon and toward the 3′UTR (Fig. 2 *A* and *B*). The methylation peaks were enriched for the DRACH consensus motif (Fig. 2*C*). GO term analysis of the methylated transcripts revealed various molecular pathways, such as gene expression regulation, RNA metabolism, neural development, and synaptic function (*SI Appendix*, Fig. S3*B* and Dataset S13).

We used the dataset generated from the mouse ACC for comparison with the human CC since both represent cortical brain regions. First, we identified all transcripts with an assigned homologue in the corresponding species, which accounted for the vast majority of all m^6^A transcripts (86% in mouse and 78% in human, Fig. 2*D*). More than half (55%) of these methylated transcripts detected in human were also methylated in the mouse ACC (Fig. 2*D* and Dataset S14). These conserved transcripts were strongly enriched for GO pathways linked to synaptic plasticity such as transsynaptic signaling, regulation of synapse organization, or learning and memory (Fig. 2*E*). Furthermore, SynGO analysis revealed that such transcripts are enrichment for synaptic location (Fig. 2*F*) and function (*SI Appendix*, Fig. S3*C*).

Further analysis revealed that also the location of methylated regions was strongly conserved since the annotated m^6^A peaks were detected in the same region of the corresponding homologous human/mouse transcripts (Fig. 2*G* and *SI Appendix*, Fig. S3*D*).

While the commonly methylated transcripts were linked to synaptic function and localization, we also detected transcripts uniquely methylated in either the human CC or the mouse ACC. The mouse-specific transcripts corresponded to genes involved with neurogenesis, the regulation of signal transmission, and synaptic function, although with considerably less significant enrichment as observed for the transcripts conserved in both species (*SI Appendix*, Fig. S3*E* and Dataset S15). Transcripts uniquely methylated in the human CC were enriched for genes associated with the regulation of gene expression, chromatin organization, and RNA metabolism (Fig. 2*H*) and did not show enrichment for synaptic localization (Fig. 2*I* and *SI Appendix*, Fig. S3*F*). In line with these observations, the experimentally confirmed synaptic transcripts were significantly enriched within the methylated transcripts detected in mice and humans. In contrast, methylated transcripts specific to humans showed comparatively low or no enrichment for synaptic mRNAs (Fig. 2*I*). Of course, care has to be taken when interpreting data comparing brain-specific gene expression between mice and humans. This is due to species differences but in some cases also due to a lack of consensus on anatomical definitions. In our study, we analyzed the CC region of the human brain corresponding to the Brodmann area (BA) 24, while it has been suggested that the ACC in mice best compares not only to human BA24 but also 25 and 36 (35). Moreover, we compared 3-mo-old mice to healthy elderly humans, and one may argue that it might be more suitable to use tissue from older mice for comparison. Therefore, we also compared data obtained from the ACC of 16-mo-old mice to the CC of healthy elderly humans which yielded similar results (*SI Appendix*, Fig. S3 *G*–*I*).

### m^6^A RNA Changes in Mouse Models of Cognitive Decline and Human AD Patient Brain Tissue.

**Fig. 3.**
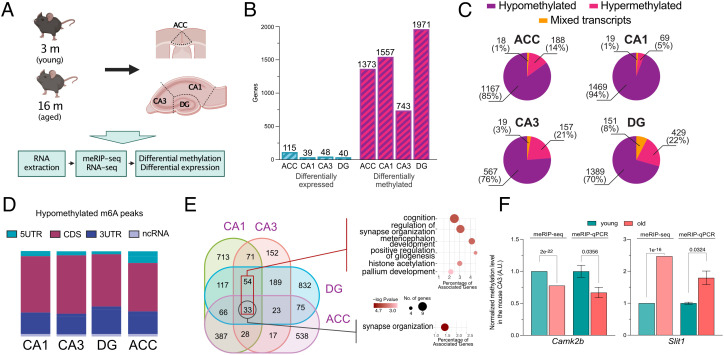
Tissue-specific m^6^A changes in the aging mouse brain. (*A*) Experimental scheme. (*B*) Bar graph showing the number of differentially expressed and differentially methylated genes detected in the corresponding brain subregion, applying equal cutoffs for FC and adjusted *P* value (FC > 1.2 and padj ≤ 0.05). (*C*) Pie charts showing the proportion of methylated transcripts containing peaks with only reduced methylation levels in aging (hypomethylated) and only increased methylation (hypermethylated) or a mixture of decreased and increased (mixed) m^6^A peaks in the analyzed brain subregions. (*D*) Bar chart showing the annotated distribution of significantly hypomethylated m^6^A peaks across transcripts for the investigated brain regions. (*E*) Venn diagram comparing the hypomethylated transcripts across hippocampal subregions and the ACC. The dark red rectangle refers to the 87 transcripts detected in all hippocampal subregions. The corresponding *Right*/*Upper* panel shows the GO term (biological process) analysis for these transcripts. The black rectangle refers to the 33 transcripts commonly hypomethylated in the hippocampus and ACC, and the *Right*/*Lower* panel shows the corresponding GO term analysis. (*F*) qPCR validation of two differentially methylated genes (hypo- and hypermethylation). The graphs show the FC in methylation as detected by meRIP-seq and meRIP–qPCR. For the meRIP-Seq side, columns show the mean FC with the FDR displayed above, as calculated by ExomePeak. For the qPCR data, the columns show the mean ± SEM of four independent replicates per condition. Statistical significance was determined by Student’s *t* test, and the *P* value is displayed above the comparison. ACC - anterior cingulate cortex, DG - dentate gyrus, 5UTR - 5′ untranslated region, 3UTR - 3′ untranslated region, CDS - coding sequence, ncRNA - noncoding RNA.

**Fig. 4.**
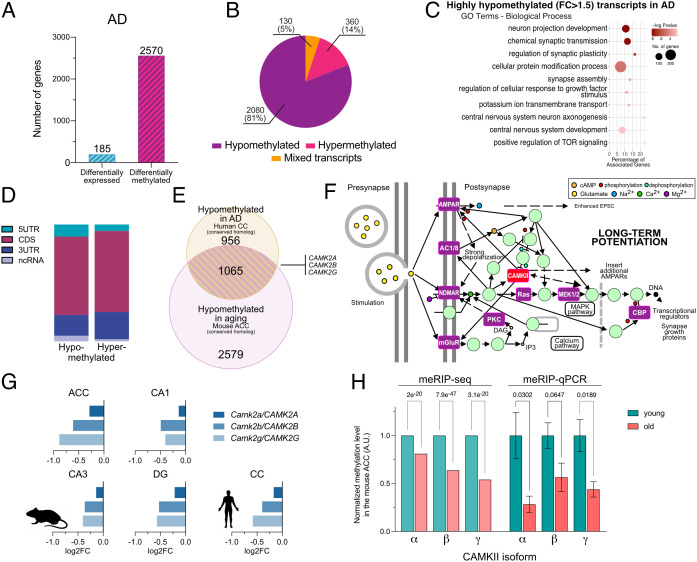
Epitranscriptomic changes in neurodegeneration and aging. (*A*) Bar plot showing the number of differentially expressed and differentially m^6^A methylated transcripts in AD vs. control samples applying equal cutoffs for FC and adjusted p value (FC > 1.2 and padj ≤ 0.05). (*B*) Pie chart showing the proportion of hypomethylated, hypermethylated, and mixed transcripts in AD samples compared to control. (*C*) Enriched GO categories (biological process) for m^6^A hypomethylated transcripts (FC > 1.5) in AD compared to control. (*D*) Bar chart showing the distribution of m^6^A peaks across transcripts hypomethylated in AD. (*E*) Venn diagram comparing significantly hypomethylated transcripts in the aged mouse ACC and CC of human AD patients. Highlighted are the CaMKII isoforms. (*F*) Image showing the KEGG pathway LTP (hsa04720). Highlighted in purple are transcripts that are commonly hypomethylated in the aging mouse ACC and human CC of AD patients. CAMKII also belongs to this group but is highlighted in red since its hypomethylation was confirmed via qPCR (*H*). (*G*) Bar plots showing the m^6^A hypomethylation for the *CamkII* isoforms compared in the young vs. aged mouse brains and the human CC in control vs. AD patients. Each bar represents a methylation site in the 3UTR of the corresponding transcript closest to the stop codon. (*H*) qPCR validation of a hypomethylated region in the 3UTR of the different *CamkII* isoforms. The graphs show the FC in m^6^A methylation detected via meRIP-seq and meRIP–qPCR. Error bars show the mean ± SEM of 6/4 (young/old) independent replicates; *P* value is displayed above the comparison. Statistical significance was evaluated by Student’s *t* test with Welch’s correction for unequal variances. AD – Alzheimer’s disease, ACC - anterior cingulate cortex, DG - dentate gyrus, 5UTR - 5′ untranslated region, 3UTR - 3′ untranslated region, CDS - coding sequence, ncRNA - noncoding RNA.

Our data support the view that the regulation of synaptic organization, function, and plasticity through m^6^A modifications might be a conserved mechanism in the adult mammalian brain. To further explore this, we studied the m^6^A landscape in a model of cognitive decline and chose age-associated memory impairment in mice as a model system. Previous studies have reported that age-associated memory impairment can be observed already in 16-mo-old mice, while at this stage, only minor changes in neuronal gene expression are detected (36–38). We reasoned that the comparison of 3- vs. 16-mo-old mice would allow us to test whether changes in m^6^A RNA methylation may precede changes in gene expression. To this end, we collected the ACC, CA1, CA3, and DG from 3- (young) and 16- (old) mo-old mice and performed meRIP-seq analysis (Fig. 3*A*). In line with previous RNA-seq data from bulk hippocampal tissue, differential gene expression analysis between samples from old and young mice revealed comparatively mild changes (FC > 1.2 and FDR ≤ 0.05) ranging from 39 differentially expressed genes (DEGs) in the CA1 to 115 in the ACC (Fig. 3*B*, *SI Appendix*, Fig. S4*A*, and Dataset S16). DEGs were not significantly enriched for any GO categories. In contrast to the transcriptome, m^6^A RNA methylation substantially differed when comparing tissue samples from young vs. old mice. Using the same cutoffs as for the differential expression analysis, 1,971 transcripts were differentially methylated in the DG, followed by the CA1 with 1,557, ACC with 1,373, and CA3 with 743 transcripts with significantly altered m^6^A modifications (Fig. 4*B* and Dataset S17). On average, 1.15 methylated regions, meaning individual peaks, were significantly altered per affected transcript (*SI Appendix*, Fig. S4). Since a transcript can carry multiple m^6^A methylation peaks, it is possible that such transcripts exhibit at the same time m^6^A peaks that increase, while others could decrease. Our data show that only in a few cases, increased and decreased m^6^A peaks were detected within the same transcript (named mixed transcripts, Fig. 3*C*). Throughout the analyzed brain regions, the majority of the transcripts characterized by altered m^6^A modifications exhibited either consistently decreased or increased changes in m^6^A methylation, which we refer to as hypo- or hypermethylated, respectively. The vast majority of significantly altered m^6^A modifications showed hypomethylation ranging from 94% in the CA1 to 70% in the DG (Fig. 3*C*). In contrast, only a small fraction of transcripts was hypermethylated (Fig. 3*C*). Interestingly, when considering the fold change (FC) and *P* value, the magnitude of hypomethylation changes across brain subregions differed, with the CA1 and ACC displaying a more pronounced reduction in m^6^A levels when compared to the CA3 and DG (*SI Appendix*, Fig. S5*A*).

The location of the m^6^A modifications within a transcript has been associated with different functional consequences. Therefore, we wondered whether the observed m^6^A methylation changes would concentrate at specific regions within the affected transcripts. Similar to the distribution observed in young mice (Fig. 1), hypomethylated peaks were detected along the gene body and showed an enrichment toward the stop codon and 3′UTR (Fig. 3*D*). Only in the ACC a slight enrichment of hypomethylated peaks within the 5′UTR was observed (Fig. 3*D* and *SI Appendix*, Fig. S5*B*). The significantly hypermethylated peaks showed a considerably larger variability in their location within mRNAs (*SI Appendix*, Fig. S5 *B* and *C*). The possibility remained that alternative splicing may confound the detected changes in m^6^A peaks. However, we could not detect any relation of the comparatively few alternative splicing events to the differences in m^6^A modifications (*SI Appendix*, Fig. S6).

When comparing the hippocampus, 87 mRNAs were commonly hypomethylated in all subregions (Fig. 3*E*), while two transcripts (*Rtn1* and *Pfn1*) were consistently hypermethylated (*SI Appendix*, Fig. S5*D*). GO term analysis of the 87 common transcripts showed that these genes are, for example, associated with cognition and synaptic organization (Fig. 3*E* and Dataset S18). Of note, differentially methylated regions detected at the sequencing level could be validated independently by meRIP–qPCR for selected transcripts (Fig. 3*F*). When further comparing the hypomethylated transcripts among the hippocampal subregions and the ACC, 33 common transcripts were detected, and GO term analysis revealed that these genes are associated with synapse organization (Fig. 3*E* and Dataset S19). While these data suggest that aging distinctly affects m^6^A RNA methylation in the hippocampal subregions and the ACC at the transcript level, the regulation of synaptic function appears to be a commonly deregulated process. This view is supported by a GO term analysis performed for each of the analyzed regions individually. Despite the limited overlap of the affected transcripts across brain regions, identical GO terms such as chemical synaptic transmission, regulation of synaptic plasticity, neurogenesis, and modulation of chemical synaptic transmission were detected when we analyzed the hypomethylated transcripts in the hippocampal regions and the ACC (Fig. 3*E*, *SI Appendix*, Fig. S5*E*, and Datasets S20–S23). In addition, many GO terms, although not identical, were related to synapse assembly that was detected in the ACC, CA1, and DG, while in the CA3 region, we found the GO term regulation of presynapse organization among the top 10 enriched processes (Datasets S20–S23). A number of GO terms were also specific to the individual hippocampal subregion. For example, GO terms linked to RNA biology including mRNA processing or mRNA splice site selection were significantly enriched when hypomethylated transcripts of the CA1 were analyzed, while these GO terms were not detected in any of the other hippocampal subregions or the ACC (*SI Appendix*, Fig. S5*E* and Datasets S20–S23).

Taken together, these data suggest that the onset of age-associated memory impairment, which is first detected in 16-mo-old mice (36, 39), is accompanied by m^6^A hypomethylation within transcripts linked to synaptic function and plasticity. Since this interpretation is based on GO term analysis, further experiments are required to study the impact of m^6^A hypomethylation on synaptic transcripts.

Next, we analyzed the m^6^A epitranscriptome in the brains of AD patients, the most common form of age-associated dementia in humans. Postmortem human cortex samples from AD patients (Braak and Braak stage IV) were matched with those of corresponding nondemented controls (NDC, Braak and Braak stage I/II) and analyzed via meRIP-seq. At the gene expression level, we detected 185 DEGs (100 up-regulated and 85 down-regulated; FC > 1.2 and FDR ≤ 0.05, Fig. 4*A* and *SI Appendix*, Fig. S7*A*). GO term analysis showed that the up-regulated genes were enriched for regulators of the Wnt signaling pathway, whereas down-regulated genes were not associated with any GO term. It is worth noting that no genes associated with the m^6^A machinery were differentially expressed in this dataset (*SI Appendix*, Fig. S7*A*).

When we performed differential methylation analysis of the meRIP-seq dataset using the same FC and FDR cutoffs as for the differential expression analysis, more than 2,500 transcripts exhibited significantly altered m^6^A modifications (Fig. 4*A* and Dataset S24). This corresponded to 3288 differentially methylated peaks, with an average of 1.26 m^6^A peaks affected per differentially methylated transcript (*SI Appendix*, Fig. S7 *B* and *C*). The majority of these changes represented hypomethylation, namely 81% of the affected transcripts were exclusively hypomethylated, while 14% showed exclusive hypermethylation. The remaining 5% of the transcripts displayed hypo- and hypermethylated regions (mixed transcripts, Fig. 4*B*). GO term analysis showed that the hypomethylated transcripts were mainly associated with neuronal function and the regulation of synaptic plasticity (Fig. 4*C* and Dataset S25). The location of the significantly altered m^6^A modifications did not favor any specific region within the affected transcripts (Fig. 4*D* and *SI Appendix*, Fig. S7*D*).

These data show some similarity to the corresponding changes observed in the aging mouse brain. There was a considerable overlap between the populations of hypomethylated transcripts in the aged mouse brain and the human AD brain, and more than 1,000 transcripts characterized by m^6^A hypomethylation were detected in both species (Fig. 4*E* and Dataset S26). GO term and Kyoto Encyclopedia of Genes and Genomes (KEGG) pathway analysis of these common transcripts showed that they play a role in the regulation of synaptic function and plasticity (*SI Appendix*, Fig. S7 *E* and *F* and Datasets S27 and S28). Especially, pathways associated with the regulation of plasticity—like long-term potentiation (LTP)—were highly overrepresented (Fig. 4*F*, *SI Appendix*, Fig. S7*F*, and Dataset S28). Furthermore, there was a significant overlap between these transcripts and previously described synaptic mRNAs, as well as synaptosomal transcripts that were found to carry m^6^A modifications (*SI Appendix*, Fig. S8) (20, 33). In addition, several key regulators of LTP could be found within this group of transcripts (Fig. 4*F*, shown in purple and red). Among them were multiple isoforms of one of the best described subfamilies of synaptic plasticity-associated proteins, the calcium/calmodulin-dependent protein kinase II (*CAMKII*), namely *C*CAMKII*a*, **CAMKII*b*, and **CAMKII*g* (Fig. 4 *E*–*G*). *CAMKII*, and especially the α and β isoforms, is central for memory formation and learning (40). The corresponding transcripts were characterized by a consistent hypomethylation in the aging mouse brain and human AD brain (Fig. 4*G* and Dataset S29). This finding was confirmed by qPCR (Fig. 4*H*) and is in line with previous reports showing that loss of the m^6^A reader YTHDF1 reduces synaptic *CAMKII* levels (21). Moreover, m^6^A RNA methylation of the **CAMKII*b* transcript was detected in the human parahippocampus (23). We also detected hypomethylated transcripts that differed between the aged mouse brain and the brain of AD patients (Fig. 4*E* and Dataset S26), which is likely due to species differences and the fact that the mechanisms underlying aging and AD are not identical. GO term analysis revealed that transcripts hypomethylated specifically in the cortex of AD patients were linked to the regulation of neuronal projection development*.* Other transcripts were linked to protein modification process, covalent chromatin modification, mRNA splicing, or intracellular protein transport (*SI Appendix*, Fig. S7*G* and Dataset S30). The transcripts specifically hypomethylated in the ACC of aging mice were linked to GO terms important for neuronal functions such as neurogenesis, regulation of nervous system development, axonogenesis, chemical synaptic transmission, and in addition to processes such as regulation of transcription by RNA polymerase II (*SI Appendix*, Fig. S7*H* and Dataset S31).

### Decreased m^6^A Levels Affect the Local Synthesis of the Plasticity-Related Protein *CAMKII*.

The fact that we see comparatively few changes in gene expression while substantial m^6^A hypomethylation is observed in aged mice and the analyzed AD brains suggests that the m^6^A changes detected in our experimental settings do not affect transcript stability, a process that has been linked to m^6^A RNA methylation (41). In line with this hypothesis, there was no obvious correlation between m^6^A modifications and expression changes in the corresponding transcript in any of the analyzed tissues (*SI Appendix*, Fig. S9 *A*–*E*). We also tested histone 3 trimethylation at lysine 36 (H3K36me3). We observed that the levels of H3K36me3, a repressive histone mark that had been linked to transcription-dependent changes in m^6^A RNA methylation (34), were similar when comparing hippocampal tissue samples from young and old mice via ChIP-sequencing (*SI Appendix*, Fig. S9 *F* and *G*). These data do not question previous findings showing that neuronal m^6^A RNA methylation regulates mRNA levels and stability (42). Although we cannot exclude the possibility that m^6^A RNA hypomethylation may affect mRNA stability in our experimental setting, our data suggest that this is unlikely the main biological consequence of the detected hypomethylation.

m^6^A modifications on mRNA are also known to play a role in the regulation of neuronal mRNA transport and translation of plasticity-related genes (14, 19, 20). To determine whether these mechanisms could be affected by m^6^A hypomethylation observed during cognitive decline, we first isolated synaptosomal compartments from the hippocampi of young and old mice and performed RNA-seq on the resulting synaptic mRNA population (*SI Appendix*, Fig. S10*A*). Similar to the analysis of bulk tissue (*SI Appendix*, Fig. S4), we detected comparatively few differentially expressed transcripts in synaptosomes when comparing 3- vs. 16-mo-old mice (three transcripts were up-regulated and none down-regulated; *SI Appendix*, Fig. S10*B*). Moreover, there was no correlation between the changes in synaptic transcript levels and the changes in their m^6^A methylation status in our experimental setting (*SI Appendix*, Fig. S10*C*). These data suggest that synaptic localization may not be the major consequence of m^6^A hypomethylation within the transcripts detected in our analysis.

Another process linked to m^6^A RNA methylation is mRNA translation (13). Thus, we performed polysome sequencing on young and old mouse hippocampal tissue samples. Differential binding analysis identified 83 genes that were differentially translated during aging (*SI Appendix*, Fig. S10 *D* and *E* and Dataset S32). However, there was no significant overlap with the transcripts affected by differential m^6^A methylation (*SI Appendix*, Fig. S10*F*).

Previous studies hypothesized that m^6^A RNA methylation plays a role in local synaptic protein synthesis (19, 23). Moreover, m^6^A was shown to control axonal protein synthesis in motoneurons (43). Thus, our analysis of bulk tissue via polysome sequencing might not be sensitive enough to detect changes in protein synthesis if specifically synaptic compartments are affected. To address this experimentally, we opted to use a primary neuronal cell culture model to evaluate the effect of reduced m^6^A levels on local protein synthesis at the synapse. Since there are no suitable high-throughput methods to assay synaptic protein synthesis, we decided to evaluate its rate and location by studying the synthesis of *CAMKII* via a puromycin-based proximity ligation assay (puro-PLA). We chose *CAMKII* since it is a well-described synaptically located transcript that is known to be synaptically translated. Moreover, *CAMKII* plays a key role in memory function (44), can undergo m^6^A modifications (23), and was hypomethylated in the aging mouse and human AD brains as shown in this study.

**Fig. 5.**
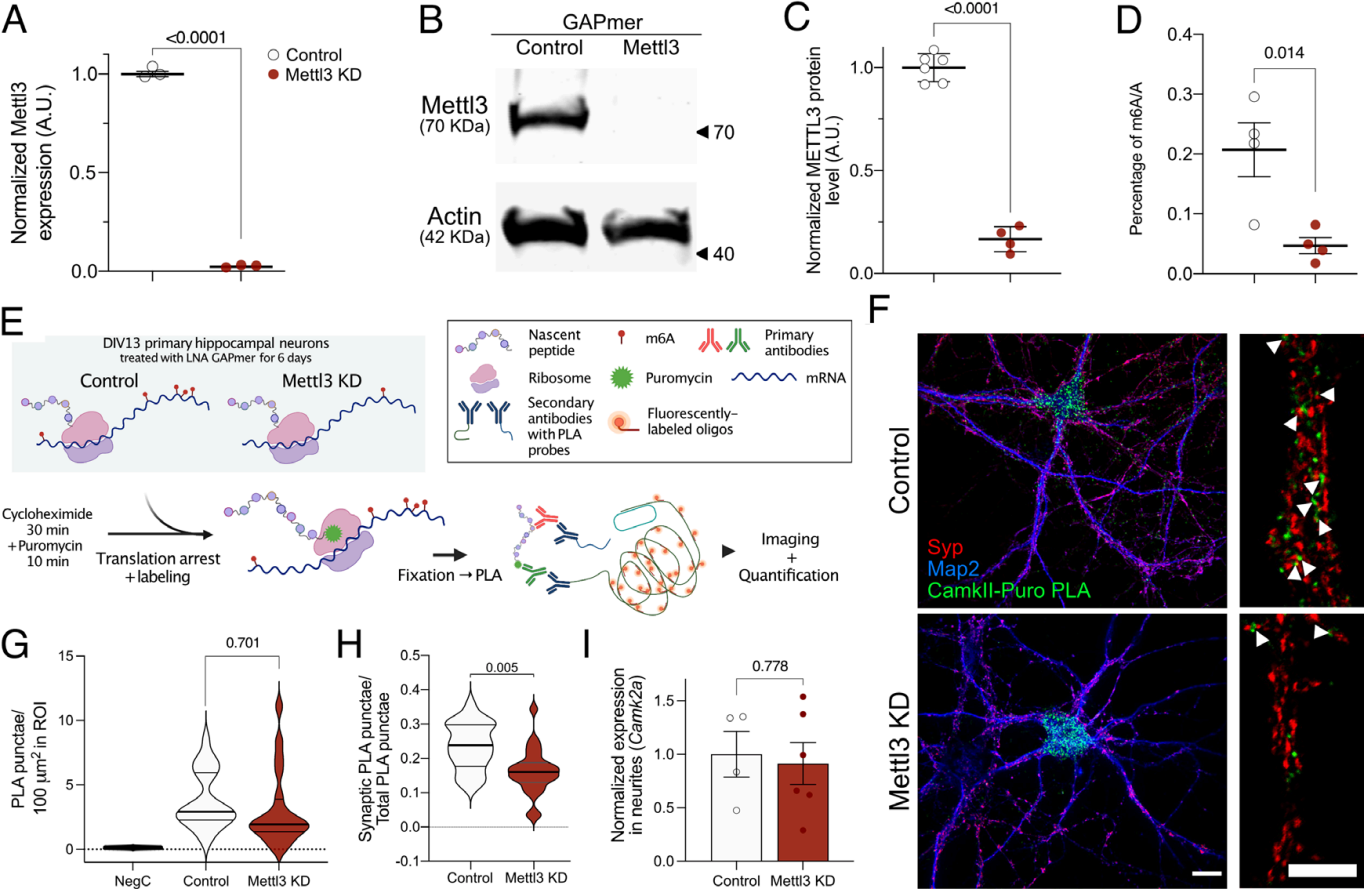
m^6^A changes influence the synaptic protein synthesis of *CAMKII*. (*A*) Bar plot showing the qPCR results for *Mettl3* expression in neuronal cultures treated with a GAPmer targeting *Mettl3* (Mettl3 KD) or a corresponding control oligonucleotide (control). (*B*) Representative immunoblot showing METTL3 protein level in response to GAPmer-mediated knockdown of *Mettl3*. (*C*) Quantification of (*B*). (*D*) Analysis of bulk m^6^A levels upon GAPmer-mediated knockdown of *Mettl3*. Graphs in *A*, *C*, and *D* display the mean ± SEM of each condition. Each data point represents one independent replicate; statistical significance was determined by Student’s *t* test. (*E*) Schematic illustration of the puro-PLA labeling used to quantify the synthesis of *CAMKII* in primary neurons. (*F*) Representative images of primary hippocampal neurons treated with either control or Mettl3 KD GAPmers. (Scale bar, 20 μm.) *CAMKII*-PLA signal is shown in green, SYP in red, and Map2 in blue. *Right* panel shows high-magnification images of a representative dendrite. (Scale bar, 10 μm.) Arrowheads indicate sites of *CAMKII* synthesis in the close vicinity of synapses. (*G*) Violin plot showing the total number of detected PLA punctae in treated neurons. Negative control (NegC) was not treated with puromycin before being processed for PLA. (*H*) Violin plot comparing the synaptically located *CAMKII*-PLA punctae in control and Mettl3 KD-treated neurons. Graphs in *G* and *H* show the mean of three independent experiments; for each experiment, 7 to 13 neurons were imaged and analyzed. Quartiles are marked by gray lines. (*I*) Normalized **CAMKII*a* mRNA levels in the synaptic compartments of primary neurons grown in microfluidic chambers. Dots in *I* represent individual independent replicates of neuronal cultures. Statistical significance was determined by Student’s *t* test. *P* values are displayed in the corresponding figure panels.

To reduce m^6^A levels, the methyltransferase *Mettl3* was knocked down (KD) in primary mouse neurons. The KD of *Mettl3* via siRNA has been reported to be challenging in primary neurons (20). Indeed, we observed only partial effects despite high concentration of siRNA probes (*SI Appendix*, Fig. S11 *A* and *B*). Therefore, we decided to employ another technology, namely LNA GAPmers, at lower doses and for longer treatment periods. Primary neurons treated with a LNA GAPmer targeting *Mettl3* packaged in lipid nanoparticles (LNPs) at day in vitro (DIV) 7 showed an almost complete reduction of *Mettl3* mRNA levels (> 95%) when measured 3 d later (Fig. 5*A* and *SI Appendix*, Fig. S11*C*). However, further 3 d of culture were necessary to sufficiently decrease METTL3 protein and m^6^A levels (Fig. 5 *B*–*D*). Having established the successful reduction of m^6^A levels, we studied the rate of protein synthesis for *CAMKII* via the puro-PLA. Puro-PLA depends on the use of the antibiotic puromycin for the labeling of nascent protein chains and N-terminal primary antibodies to detect sites of translation through proximity ligation (Fig. 5*E* and *SI Appendix*, Fig. S11*D*) (45). A cycloheximide pretreatment was also applied to improve the spatial localization of sites of protein synthesis (46). Puromycin labeling and translational arrest were confirmed in neurons (*SI Appendix*, Fig. S11*E*). DIV 13 primary neurons that had been treated at DIV 7 with either a *Mettl3* KD or control GAPmer were processed for puro-PLA using an antibody that detects the N terminus of *CAMKII* α, β, and γ (Fig. 5 *E* and *F*). Puro-PLA–treated neurons were imaged by a confocal microscope, and the PLA punctae were automatically detected and quantified. The synaptic marker synaptophysin (SYP) was used to determine the synaptic localization of the detected PLA punctae (Fig. 5*F*). Neurons with reduced levels of m^6^A (Mettl3 KD) showed a reduction of PLA punctae in dendritic projections (Fig. 5*F* and *SI Appendix*, Fig. S12*A*). Quantitative analysis revealed that the total number of PLA punctae in the whole neuron was not significantly reduced (*SI Appendix*, Fig. S12*B*). The number of synapses detected via SYP staining was also not significantly changed in response to decreased m^6^A levels (*SI Appendix*, Fig. S12 *C* and *D*). However, when looking at the proportion of *CAMKII*-PLA punctae detected in the vicinity of SYP+ synaptic compartments, the Mettl3 KD-treated neurons showed significantly decreased numbers (Fig. 5 *F* and *H*). We furthermore confirmed that the reduced local translation of *CAMKII* resulted in reduced synaptic protein levels (*SI Appendix*, Fig. S13 *A* and *B*). Although our analysis of synaptosomes in young and aged mice indicated that changes in m^6^A modifications do not affect the total levels of synaptic transcript (*SI Appendix*, Fig. S10), we wanted to further test the possibility that the differences observed in *Mettl3* KD-treated neurons might be a consequence of decreased mRNA transport to synaptic compartments. We used a previously established custom-made microfluidic chamber culture system to isolate synapse-localized transcripts (*SI Appendix*, Fig. S2*E*) (33). *Mettl3* KD treatment on the somas of the cultured neurons showed no significant effect on the amount of **CAMKII*a*, **CAMKII*b*, and **CAMKII*g* mRNA located in synaptic compartments (Fig. 5*I* and *SI Appendix*, Fig. S12*E*). To confirm that the effect of reduced m^6^A modifications on synaptic mRNA translation is not exclusive to *CAMKII*, we performed puro-PLA for another key synaptic protein, which we also found to be hypomethylated in aging and AD. We selected GLUA1, which is known to be locally synthesized and has been linked to aging and neurodegenerative diseases (47). Synaptic GLUA1 synthesis was significantly decreased in *Mettl3* KD neurons when compared to control (*SI Appendix*, Fig. S14). In line with these data, we found that *Mettl3* KD-mediated loss of m^6^A RNA methylation impaired the network activity of neurons in culture, which is highly dependent on synaptic function and plasticity when measured via a multielectrode array (*SI Appendix*, Fig. S13).

## Discussion

In this study, we aimed to dissect the role of m^6^A RNA methylation in the brain and analyzed the m^6^A epitranscriptome in mice and humans in the context of cognitive decline associated with aging and AD. We found that 40 to 44% of all transcripts in the different hippocampal subregions in mice carried m^6^A modifications. These data are in line with recent studies in which different brain regions or bulk hippocampal tissue had been analyzed for m^6^A RNA methylation (30, 48, 49). m^6^A-labeled mRNAs in all of the hippocampal subregions were strongly enriched for genes associated with the regulation of synaptic function and plasticity. Moreover, 60% of the transcripts with m^6^A modifications were common to all hippocampal subregions. Similarly, when comparing the ACC and hippocampus, 61% of the m^6^A-modified transcripts could be detected in both brain regions. These results are in agreement with previously published data comparing m^6^A-modified transcripts across different tissues (16, 48). The commonly m^6^A-modified transcripts across brain regions were mainly involved in synaptic function and structure and were overrepresented for mRNAs that are localized to synapses. These data are in line with previous findings suggesting that m^6^A transcripts are detected at synapses (20, 23) and support the view that m^6^A-dependent mechanisms play an important role in synaptic plasticity. Our data also support a recent study in which a reporter transcript was fused to the 3′UTR of selected synaptic RNAs such as **CAMKII*a* to show that synaptic localization is reduced when the adenosine of the m^6^A sites within the corresponding 3′UTR region is changed for a guanine (50).

Interestingly, processes linked to synaptic function were not among the most significant GO terms when m^6^A transcripts specific to the individual hippocampal subregions were analyzed. For example, protein complex assembly, nuclear export, and ncRNA processing were the most significant GO terms associated with m^6^A transcripts specific to the CA1, CA3, and DG regions, respectively. These data suggest that the m^6^A transcripts specific to the hippocampal subregions represent distinct molecular processes. However, further research is required to fully understand the molecular and cellular consequences of this observation. This is also true for the comparison of m^6^A transcripts in the mouse ACC and hippocampus. While the transcripts specific to the hippocampus could not be linked to any significant GO term, the most significant GO terms for m^6^A transcripts specific to the ACC were associated with chemical synaptic transmission. These data may suggest that synaptic processes in the ACC are even more tightly controlled via m^6^A RNA methylation when compared to the hippocampus.

When comparing the m^6^A landscape of the mouse ACC and human CC, we observed that 56% of the methylated transcripts found in humans were also detected in mice. This is remarkable when considering that a similar degree of conservation is observed when the ACC is compared to the hippocampus within the same species (61% in mice). These data are also in agreement with a previous study reporting a 62% overlap between m^6^A-modified transcripts in the mouse and human cerebella (48). The commonly methylated transcripts detected in the mouse and human cortices were mainly linked to the regulation of synaptic function and plasticity and showed a strong overrepresentation of transcripts found at synapses. Interestingly, the m^6^A transcripts specific to mice were also enriched for GO terms related to synaptic plasticity, while the methylated transcripts specific to the human CC were also enriched for GO terms linked to RNA processing and gene expression control. These data may suggest that the m^6^A-mediated orchestration of synaptic plasticity could be an evolutionary conserved mechanism in mammals, while a role of m^6^A RNA modification in gene expression control became specifically important in the human brain. This is however speculative, and care must be taken when performing these kinds of interspecies comparisons also because of the complex relationships between homologous brain regions in mice and humans (35).

To study the epitranscriptome in the context of cognitive decline, we analyzed the brains of aging mice and human AD patients. During aging, the onset of significant cognitive impairment is believed to represent the transition between normal aging and pathology (51, 52). Previous data demonstrated that mice start to display first memory impairment at 16 mo of age (36, 53). When we compared 3- vs. 16-mo-old mice, we observed a massive m^6^A hypomethylation across multiple transcripts in all investigated brain regions. This was accompanied by comparatively mild changes in gene expression, which is in agreement with previous studies showing that only minor changes in gene expression are observed in the hippocampus and cortex when comparing 3- vs. 16-mo-old mice (36). Our findings are also in line with observations from other postmitotic and excitable tissues, namely the heart. Here, during the pathogenesis of heart failure, massive changes in m^6^A hypomethylation preceded changes in gene expression (54).

While about 60% of the m^6^A-modified transcripts were common to all investigated brain regions in 3-mo-old mice, aging had more specific effects on the epitranscriptome within the individual hippocampal subregions and the ACC. Yet, the comparatively few commonly hypomethylated transcripts were all associated with synapse function. Thus, our data suggest that the different brain regions undergo distinct changes in m^6^A RNA methylation during aging, while the GO term analysis revealed that many transcripts specifically altered in the analyzed brain regions are similarly linked to processes directly associated with synaptic function. Interestingly, processes related to RNA regulation such as mRNA processing or mRNA splice site selection were only detected in the CA1 region of aged mice which might indicate that gene expression control in the CA1 region is most sensitive to the aging process and under tight control of m^6^A RNA methylation. Thus, it would be interesting to see whether gene expression changes would be more severe in the CA1 region when mice older than 16 mo are analyzed. Future research should address this question.

In summary, these data suggest that loss of m^6^A RNA methylation coincides with the onset of memory impairment in the aging brain and may contribute to cognitive decline. This view is supported by previous data showing that a knockdown of the m^6^A demethylase FTO in the prefrontal cortex of mice results in an improved consolidation of fear memories (42). Similarly, loss of the m^6^A reader YTHDF1, which has been linked to enhanced translation, leads to impairment of hippocampal LTP and memory formation in mice (21).

Another recent study analyzed m^6^A levels in the brains of 2-wk-old and 1-, 1.5-, 6.5-, and 13-mo-old mice. In comparison to our data, the authors observe comparatively milder changes, and the affected transcripts were mainly characterized by increased m^6^A modifications within the UTR when comparing 1.5- vs. 13-mo-old mice (30). These data are however difficult to compare since 1.5-mo-old mice could still be considered juvenile. Moreover, animals at 13 mo of age do not exhibit detectable memory impairment when compared to their younger counterparts (53). Longitudinal studies in mice showed that memory impairment manifests between 15 and 16.5 mo of age (53). It is therefore also possible that the increased m^6^A RNA modification observed when comparing 1.5- vs. 13-mo-old mice represents compensatory mechanisms. In line with this interpretation, the affected genes were linked to pathways such as cellular stress signaling (30). The same study also analyzed m^6^A levels in the brains of 6-mo-old 5xFAD mice, a mouse model for amyloid deposition. Here, decreased m^6^A levels were observed when comparing WT to 5xFAD mice, and the affected transcripts were linked to GO terms such as synaptic transmission (30). Since previous data showed that 5xFAD mice start to display memory impairment around 6 mo of age (55), these data are in agreement with our observations. Nevertheless, more research is needed to elucidate the dynamics of m^6^A modifications across the transcriptome of the aging and diseased brains.

Additional support for the hypothesis that cognitive decline is accompanied by m^6^A hypomethylation of transcripts important for synaptic function stems from our observation that m^6^A-modified transcripts display massive hypomethylation in the postmortem human cortex of AD patients. Moreover, there was a significant overlap between the hypomethylated transcripts in the aging mouse ACC and the human CC of AD patients. In line with this observation, the commonly hypomethylated mRNAs were associated with processes such as synaptic plasticity, LTP, or multiple pathways linked to neurodegeneration. These data are in line with the fact that cognitive aging is an important risk factor of AD and that both processes exhibit similarities, such as synapse loss, inflammation, or oxidative stress, which have led to the hypothesis that AD reflects—at least in part—accelerated aging (56). Nevertheless, several hypomethylated transcripts were specifically altered in either the aging mouse brain or in human AD patients. Although the transcripts differed, GO term analysis revealed that processes linked to neuronal function and gene expression control were affected in aging mice and in AD patients. Other GO terms such as chromatin modification were specific to humans. This is likely due to the anatomical differences between species (35) but may also reflect the fact that, besides the abovementioned similarities in age-associated cognitive decline and AD, the course of AD differs from physiological brain aging at various levels including distinct neuropsychological changes and specific gray and white matter alterations (57) (58).

The finding that AD is associated with m^6^A hypomethylation is in agreement with recent studies showing decreased mRNA and protein levels of the m^6^A methyltransferase METTL3 in hippocampal and cortical tissues of AD patients (24, 27). Reduced expression of m^6^A writers could indeed be one mechanism to explain lower levels of m^6^A RNA modification in AD. In line with this view, knockdown of *Mettl3* exacerbated Tau pathology in a Drosophila model for AD (39) and neurodegenerative phenotypes in a mouse model for amyloid deposition (27). It should be mentioned that the role of m^6^A RNA methylation in neurodegenerative disease may be more complex. For example, a recent study observed increased m^6^A levels in a mouse model for Tau pathology and in the brains of human AD patients (35). However, these data are based on a semiquantitative analysis of m^6^A immunostaining within the soma, which is difficult to compare to sequencing-based approaches.

Similarly, another recent study reported an increase in bulk m^6^A and METTL3 levels, while FTO protein levels were decreased in the hippocampus and cortex of 9-mo-old APP/PS1 mice (28). The fact that the analysis of sequencing based vs. bulk m^6^A levels currently appear contradictory may indicate that there is an RNA species, which undergoes hypermethylation in neurodegenerative diseases, that is not captured by the current sequencing approaches but dominates the analysis of bulk m^6^A levels. For example, recent evidence hints to an important role of m^6^A methylation of pre- and mature microRNAs (59). It will be interesting to study microRNA methylation in brain diseases.

In addition, it will be important to study m^6^A modifications in neuronal subcompartments. In fact, in our experimental settings, m^6^A hypomethylation occurred mainly within synapse-localized transcripts, pointing to a role of m^6^A RNA methylation in the synaptic translation of mRNAs, a well-known phenomenon that ensures the supply of key proteins necessary for synaptic function and plasticity in response to stimuli (60, 61). The function of m^6^A in the regulation of local protein synthesis has been shown in the axons of motoneurons (43), and recent data suggest a role in synaptic protein synthesis (23). We analyzed the synaptic protein synthesis of two transcripts that code for key regulators of synaptic plasticity and undergo m^6^A hypomethylation in the aging mouse brain and in the brains of AD patients, namely *Camk2* and *Glua1*. *Camk2* was also among the list of transcripts that underwent hypomethylation in the cortex of 6-mo-old 5xFAD mice (30). Our results showed that m^6^A RNA hypomethylation achieved by the knockdown of *Mettl3* expression significantly impaired synaptic translation of *Camk2* and *Glua1* mRNAs*.* In line with this observation, knockdown of *Mettl3* was associated with decreased neuronal activity. These data support the view that a m^6^A-dependent mechanism orchestrates synaptic protein synthesis and contributes to impaired synaptic function when deregulated. To further substantialize this view, future research is needed. For example, it would be important to explore the role of m^6^A hypomethylation in synaptosomes of young and aged mice and in AD models via a combination of meRIP-seq and Ribo-Seq methods. It is also important to reiterate that in addition to local mRNA translation, m^6^A modification of neuronal transcripts was shown to affect other processes such as mRNA stability (42). Since in our experimental settings, changes in m^6^A levels exceeded by far changes in transcript levels, mRNA stability does not seem to be the major process affected in the aging mouse brain in the analyzed human AD brains. However, we cannot exclude the possibility that process such as mRNA stability will be affected by changes in m^6^A levels when time points or different brain regions are analyzed.

More research is also needed to further elucidate the exact mechanism by which m^6^A levels control the synaptic translation of mRNA transcripts and better understand the processes that underlie decreased in m^6^A RNA methylation during aging and in AD. In this context, it is noteworthy that the m6A demethyltransferase FTO was shown to be present in cytoplasmic regions near to synapses (17) but decreased in protein abundance during learning (42). At the same time, the m^6^A reader YTHDF1 is also located in synaptic compartments, and its protein levels increase significantly following fear conditioning in the hippocampus (21). Furthermore, immunohistochemical analysis suggests that NMDA or KCL treatment of differentiated neuroblastoma or medulloblastoma cells enhances the synaptic colocalization of the m^6^A signal with YTHDF1 (23). YTHDF1 was shown to promote translation (21). This might be a mechanism by which a reduction of m^6^A RNA modifications affects synaptic protein synthesis. Consistently, the knockdown of *Ythdf1* was shown to negatively affect spine formation, LTP, and hippocampus-dependent learning in mice (21). In addition, recent data also implicate the m^6^A reader YTHDF3 and the m^6^A eraser ALKBH5 with the regulation of m^6^A RNA methylation at the synapse providing additional evidence for a key role of m^6^A modifications in synaptic plasticity (23). Of course, other m^6^A readers may also play a role in the regulation of synaptic mRNA translation via processes like mRNA degradation, transport, or phase separation, as proposed by several previous studies (20, 23, 26). In summary, changes in the localization and function of m^6^A writers, readers, and erasers may underlie the deregulation of m^6^A RNA methylation observed during aging and AD. In addition, metabolic changes may contribute since the methyl donor S-adenosyl methionine (SAM) was shown to be decreased in the brains of AD patients (62). Moreover, a meta-analysis of AD mouse models treated with SAM-supplemented food confirmed the beneficial effects on cognitive function (63). It will be interesting to investigate whether this effect is linked to m^6^A RNA methylation.

In conclusion, our data provide an important resource to the field and further elucidate the function of m^6^A RNA modification in young and aged mouse brains and in the brains of cognitively intact humans and AD patients. Since decreased m^6^A RNA methylation of synaptic genes is observed in brain aging and in AD, targeting the m^6^A RNA methylation machinery might be a promising strategy to prevent cognitive decline.

## Materials and Methods

### Human AD Tissue.

A total of 12 postmortem samples from the ACC (BA 24) were obtained from the Netherlands Brain Bank (NBB). The samples corresponded to six diagnosed AD patients (age 89.33 ± 4.42 y, Braak and Braak stage IV, and postmortem delay (PMD) 6:34 ± 1:00) and 6 NDC (age 86.33 ± 3.25 y, Braak and Braak stages I–II, and PMD 6:16 ± 1:38). Braak and Braak staging is an established approach to define the degree of AD pathology. While stage I/II refers to early stages with altered pathology in the brain stem, at stage IV, cortical regions are affected (64). All individuals, except one AD patient, were female. All experiments were approved by an ethics committee.

### meRIP.

RNA samples were processed as previously described for meRIP-seq (54).

### Library Preparation and Sequencing.

Samples were prepared for sequencing using the TruSeq Stranded Total RNA Library Prep Kit (Illumina) or the SMARTer Stranded Total RNA-Seq Kit v2—Pico Input Mammalian (Takara) according to the manufacturer’s instructions. For more information, see *SI Appendix*, *Supplementary Methods*. Additional metadata is also available via the GEO database (GSE198526).

### Bioinformatic Analysis of meRIP-seq and RNA-seq.

Raw reads were processed and demultiplexed using bcl2fastq (v2.20.2), and low-quality reads were filtered out with Cutadapt v1.11.0 (65). Filtered reads were mapped to the human (hg38) or mouse (mm10) genome using the STAR aligner v2.5.2b (66). The resulting bam files were sorted and indexed, and the unmapped reads removed using SAMtools v1.9.0 (67). Methylation sites were determined using MeTPeak v1.0.0 (68), and differential methylation (hypo- and hypermethylated regions) was assessed with ExomePeak v2.16.0 (69) using young samples as control and old as treatment for the mouse data, while in the case of human data, healthy individuals were used as control and AD patients as treatment. An adjusted *P* value (padj, also termed as FDR [false discovery rate]) cutoff of 0.05 and FC cutoff of 1.2 or 1.5 were used as indicated in the text. For mouse samples, only consistently significantly differentially methylated peaks were used, unless indicated; for human samples, significantly differentially methylated peaks were used.

For RNA-seq analyses, read counts were obtained with subread’s featureCounts v1.5.1 (70) from the bam files of input samples. Differential gene expression was determined by DESeq2 v3.5.12 (71) using normalized read counts and correcting for covariates detected by RUVseq v1.16.1 (72). Cutoffs of padj ≤ 0.05, FC ≥ 1.2, and BaseMean ≥ 50 were applied to the results. Background expressed genes were determined for each region as those genes with a BaseMean > 50 in the corresponding input sample.

For visualization, bam files of both IP and input samples were collapsed for PCR duplicates using SAMtools, and IP samples were normalized to their corresponding inputs and to their library size using deeptools’ v3.2.1 (73) bamCompare. The resulting normalized tracks were visualized in the IGV Browser 2.9.2 (74).

### GO Analysis.

GO term enrichment analyses were performed using the App ClueGO v2.5.3 in Cytoscape 3.7.2 (75), with GO Term Fusion enabled to collapse terms containing very similar gene lists and using a custom background corresponding to expressed genes in the corresponding species as obtained from RNA-seq results of the corresponding input samples of the meRIP experiments. GO term tables for biological process, cellular component, pathways, and KEGG were produced and are labeled accordingly in the figures. Resulting enriched GO terms were visualized with a custom script using ggplot2 v3.3.5 (76) displaying the adjusted p value (padj) for the GO term, the number of genes from the list that belong to said term, and the percentage of the total genes in the GO term that are present in the list. Synaptic GO enrichment analyses were performed with SynGO (v1.1, syngoportal.org) (32).

### Additional Bioinformatic Packages and Tools.

Scripts and analysis pipelines were written in R (3.5.2) (77). Peak annotation was performed with Homer v4.10.4 (78) and Annotatr v1.8.0 (79). Guitar plots were produced with the Guitar v1.20.1 (80) R package. Volcano plots were generated with plot.ly/orca v4.9.4.1 (81). Area-proportional Venn diagrams were produced with BioVenn (www.biovenn.nl), and multiple list comparisons performed with Intervene/UpSet (asntech.shinyapps.io/intervene/). Mouse/human homologues were determined by their annotation in NCBI’s HomoloGene database using the HomoloGene (v1.4.68.19.3.27) R package. Odds ratios and p values to determine significance in overlapped datasets were calculated with the GeneOverlap R package v1.18.0 (82). De novo motif analyses were performed with Homer’s findMotifsGenome, and motifs containing the DRACH consensus sequence out of the top 10 most significant are displayed. KEGG pathway enrichment was produced with KEGG Mapper (www.genome.jp/kegg/mapper/) (83). Microscopy images were preprocessed with Fiji, and quantification was automated in Cell Profiler (cellprofiler.org) (84). Graphs, heat maps, and statistical analyses were performed on GraphPad Prism version 9.3.1 for Mac. Some custom figures were created with BioRender (biorender.com).

### qPCR.

qPCR was performed as described before (53). Primer sequences are available as Dataset S33.

### Hippocampal Primary Neuronal Culture.

Primary neurons were prepared as described recently (53).

### Western Blot/Immunofluorescence.

Antibodies used for western blot, immunofluorescence, and other applications, as well as the dilutions used, are described in Dataset S33.

### Puro-PLA.

Puromycin-based proximity ligation assay (puro-PLA) was performed as previously described with minor alterations (45). DIV 13 mouse primary hippocampal neurons were pretreated with 100 ug/mL cycloheximide for 30 min to arrest translational elongation. Cells were then treated with 3 μM puromycin for 10 min to label nascent polypeptide chains. This treatment time was chosen to balance labeling intensity with the propensity of labeled peptides to diffuse away from their synthesis sites (46, 85). Puromycin incorporation and cycloheximide pretreatment were validated by western blot.

### Polysome Sequencing.

Polysomes were prepared from the DG of five young and five old animals as described previously (54).

### Synaptosome Isolation for Sequencing.

Synaptosomes were isolated from the hippocampi of 3- and 16-mo-old mice as recently described (33).

### H3K36me3 ChIP.

Cell-type–specific chromatin isolation and ChIP sequencing were performed as previously described (86). 3 CA1 were pooled for each replicate, and nuclei were FACS sorted by NeuN expression. 300 ng of chromatin and 1 µg of H3K36me3 antibody (Abcam, ab9050) were used for each ChIP.

## Supplementary Material

Appendix 01 (PDF)

## Data Availability

GEO database: GSE198526. Datasets S1–S33 can be accessed via the following link: https://doi.org/10.6084/m9.figshare.21966983.v1 (87).
