# Supplementary material for: Conserved reduction of m6A RNA modifications during aging and neurodegeneration is linked to changes in synaptic transcripts
Source: Proc Natl Acad Sci U S A. 2023 Feb 22;120(9):e2204933120. doi: 10.1073/pnas.2204933120 (PMC9992849; doi:10.1073/pnas.2204933120)
Supplement: Supplementary file 1 — Appendix 01 (PDF) [file pnas.2204933120.sapp.pdf]

Supplemental Material

Supplemental Figure 1

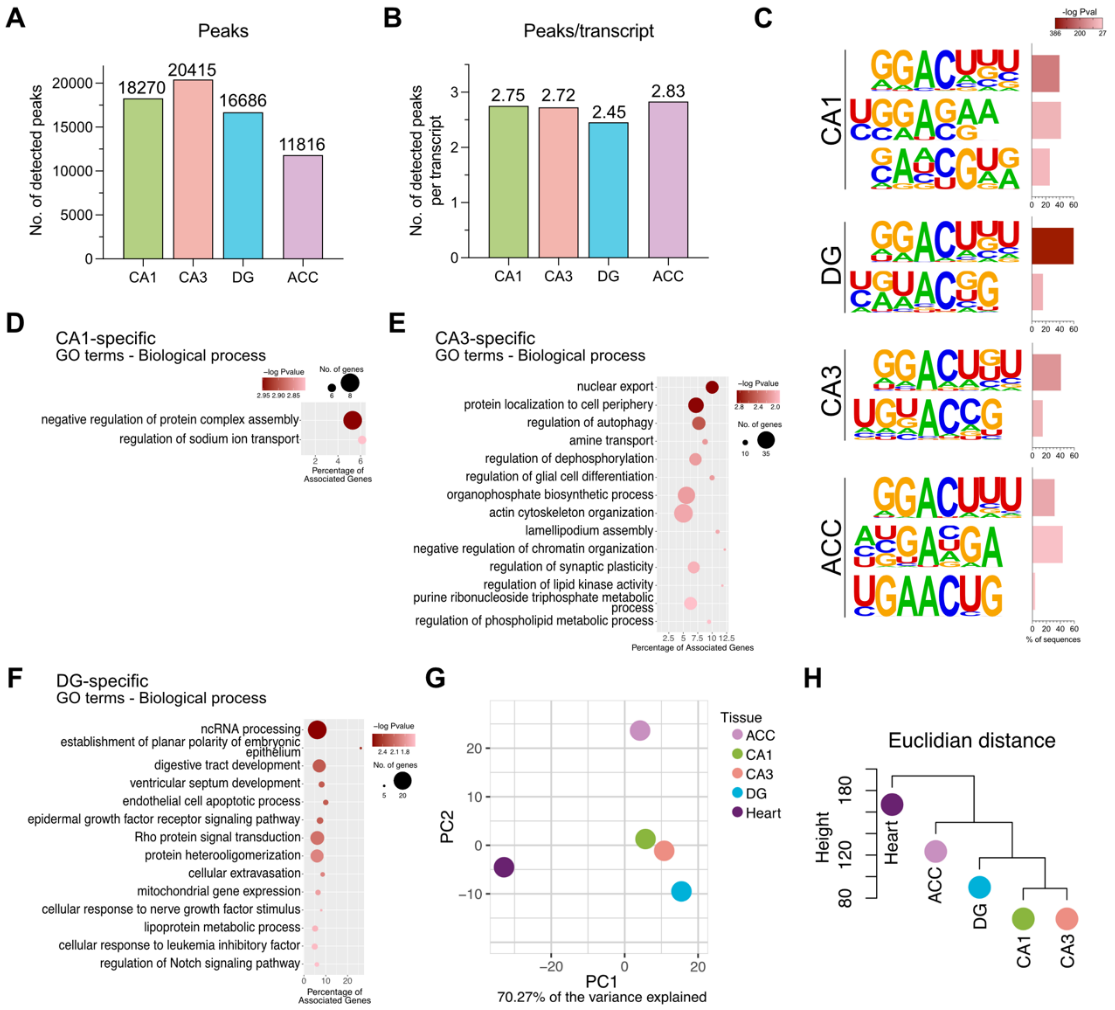

Figure S1. m^{6}A in the brain of 3 months old mice A. Bar plot showing the m^{6}A peaks detected in the hippocampal subregions and the ACC. **B.** Average number of m^{6}A peaks detected per methylated transcript. Exact values are displayed above the corresponding bar. **C.** Motif analysis identifies the DRACH m^{6}A RNA methylation consensus sequence amongst the top enriched motifs in m^{6}A peaks. **D-F.** Enriched GO terms (Biological processes) for m^{6}A methylated transcripts detected in the CA1- (D), DG- (E) and CA3 (F) regions. **G.** PCA showing PC1 and PC2 for the m^{6}A methylation level of all detected peaks in the ACC, CA1, CA3 and DG, as well as in in the heart (dataset published by (1)). **H.** Euclidean distance clustering showing the distances between the methylation levels of all detected methylation sites across all tissues.

Supplemental Figure 2

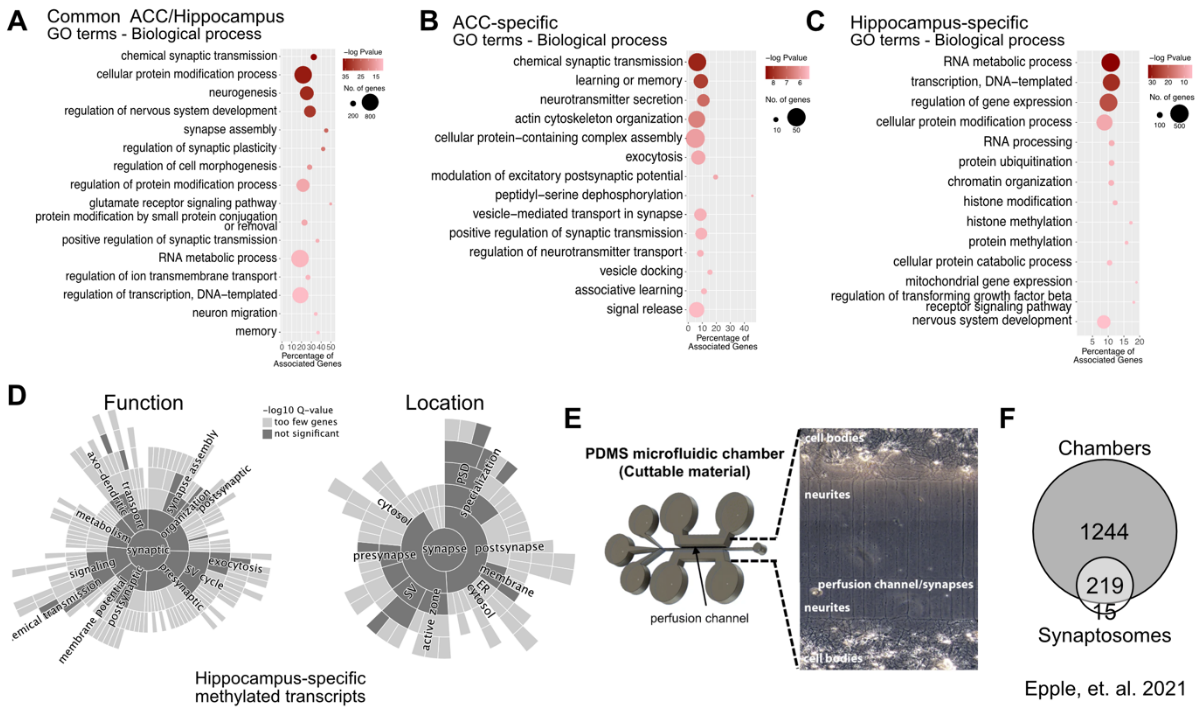

Figure S2. Characteristics of the m^{6}A transcripts detected in the brain of 3 months old mice. A-C. Enriched GO terms (Biological process) for m^{6}A methylated transcripts common between the hippocampus and the ACC (A), specific to the ACC (B) or specific to the hippocampus (C). **D.** Sunburst plots showing that synaptic GO-terms are not enriched in hippocampus-specific m^{6}A methylated transcripts**. E.** Diagram showing the microfluidic chambers used by Epple, et. al (2021) (2) to isolate synaptic RNAs. **F.** Summary of the synaptic transcriptome as described by (2).

Supplemental Figure 3

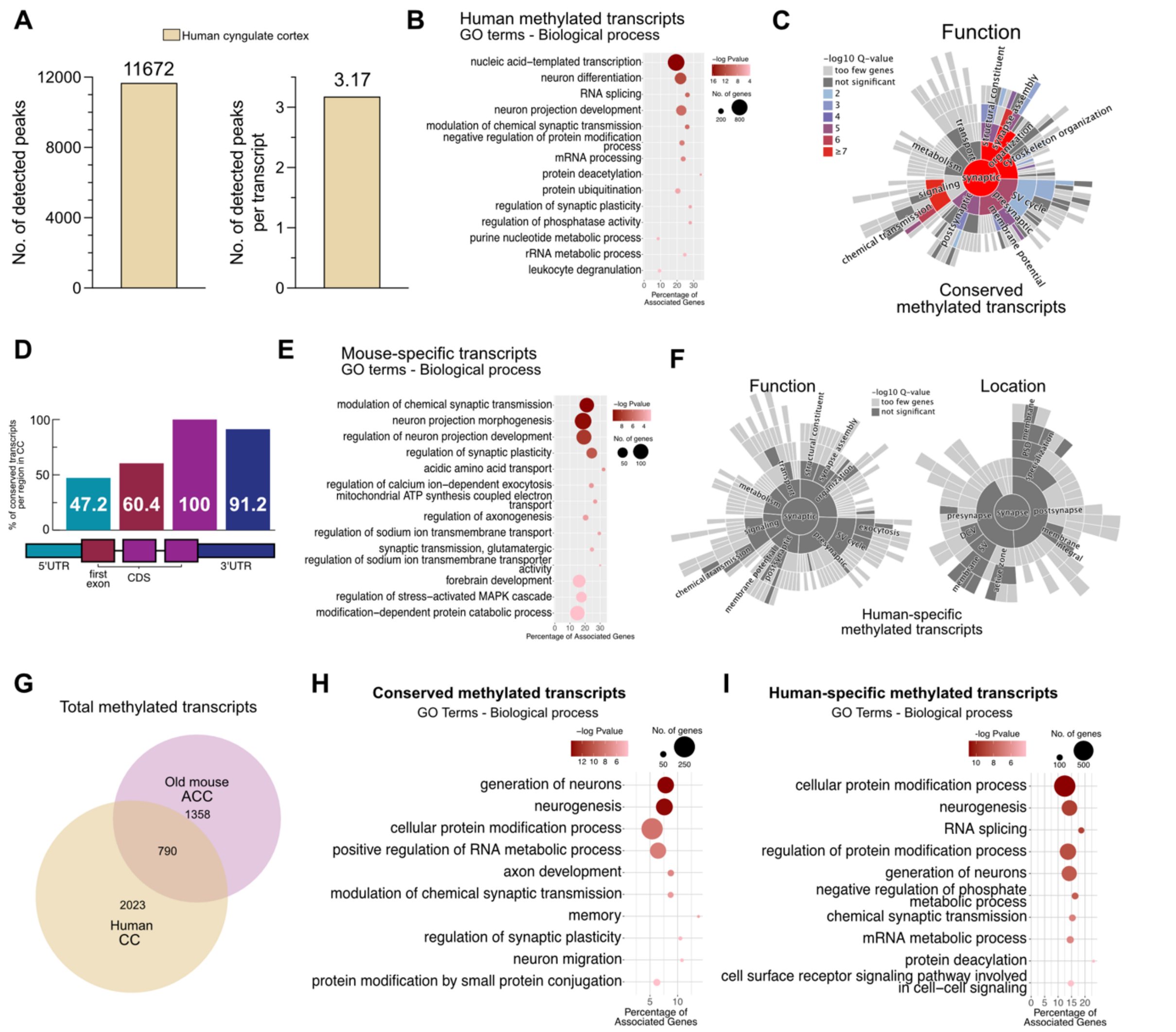

Figure S3. Characteristics of the m^{6}A transcripts detected in the human cingulate cortex. A. Bar plot showing the number of detected m^{6}A peaks (left panel) and average number of peaks per methylated transcript (right panel) in the human CC. **B.** Enriched GO terms (Biological process) for all m^{6}A methylated transcripts in the human CC. **C.** Sunburst plot showing the enriched SYNGO GO-terms for m^{6}A methylated transcripts conserved in mouse ACC and human CC. **D.** Bar plot showing the distribution of m^{6}A methylation sites across transcripts that are conserved in mice and humans. Bars show the percentage of peaks annotated for a given region in the human CC that have a corresponding peak annotated to the same region in the mouse homolog. 5'UTR, first exon, CDS and 3'UTR. **E.** Enriched GO terms (Biological process) transcripts exclusively from the ACC. **F**. SYNGO GO terms showing the lack of significant enrichment for synaptic processes when human-specific m^{6}A methylated transcripts are analyzed. **G.** Venn diagram comparing m^{6}A methylated transcripts between the aged mouse ACC and the human CC from control individuals. Please note that m^{6}A RNA

methylation is significantly decreased in the aged mouse ACC (see Figure 3), which could explain the fact that the number of commonly m^6A methylated transcripts is lower in this comparison (see also Figure 2D). **H.** Enriched GO terms (Biological process) for the commonly m^6A methylated transcripts described in (G). **I.** Enriched GO terms (Biological process) for m^6A methylated transcripts exclusive to the human CC as shown in (G).

Supplemental Figure 4

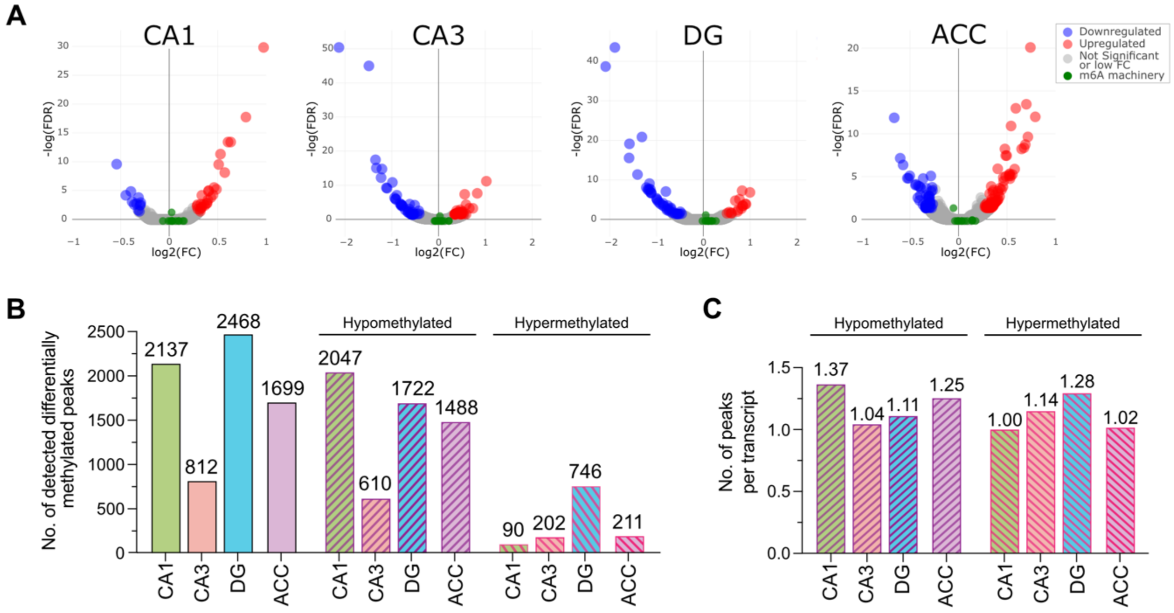

Figure S4. Changes in gene-expression and m^{6}A RNA methylation in the aging mouse brain. A. Volcano plots displaying the changes in gene expression across brain subregions comparing 3 vs 16 months old mice. Cutoffs for significance are FC > 1.2 and FDR ≤ 0.05. Highlighted in green are the known m^{6}A writers, readers and erasers, showing that no m^{6}A associated protein is differentially expressed in the aged brain. The number of differentially expressed genes are 39 for CA1, 48 for CA3, 40 for DG and 115 for ACC. **B.** Bar graphs showing the total amount differentially methylated peaks (FC > 1.2, FDR ≤ 0.05) in the brain subregions and how many of them are hypo- and hypermethylated. **C.** Bar graphs showing the number of m^{6}A peaks per differentially methylated transcript. Numbers above the bars display the exact number of peaks. The average number of peaks changing per transcript is 1.15.

Supplemental Figure 5

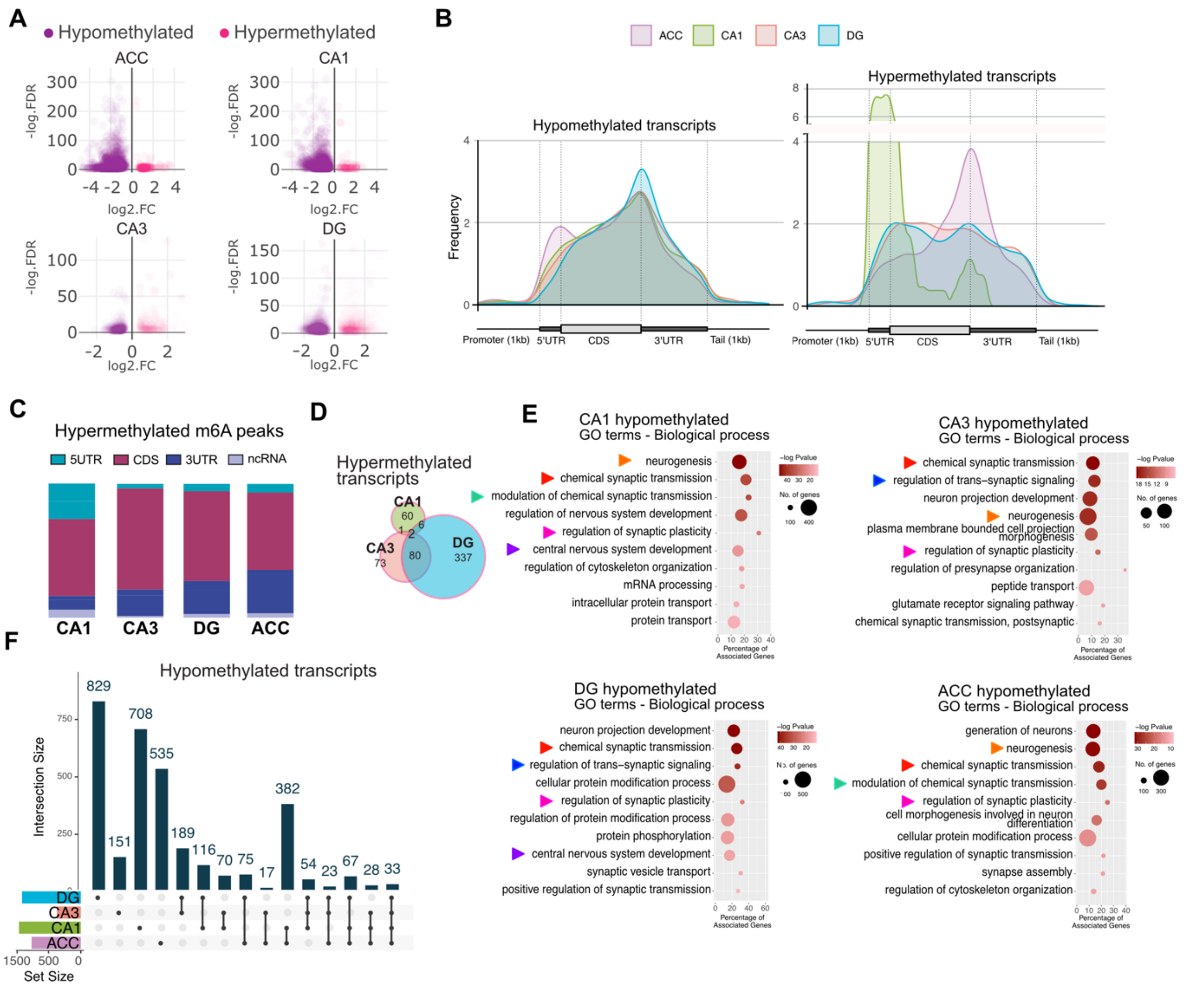

Figure S5. m^{6}A RNA methylation in the aging mouse brain. A. Volcano plots showing the magnitude of m^{6}A RNA methylation changes within the different brain regions when comparing 3 vs 16 months old mice. **B.** Guitar plots showing the frequency of hypomethylated (left panel) and hypermethylated (right panel) m^{6}A peaks along mRNA transcripts within the investigated brain regions. Please note that the plot for hypermethylated transcripts should be interpreted with care, due to the comparatively low number of affected transcripts. **C.** Distribution of hypermethylated peaks along mRNA transcripts within the investigated brain regions. **D.** Venn diagram comparing m^{6}A hypermethylated transcripts between hippocampal subregions. **E.** GO term (biological process) analysis for m^{6}A transcripts exclusively hypomethylated to the indicated brain region. Colored arrows point to GO terms detected in more than one of the analyzed brain regions. **F.** Intersect graph showing the overlap between hypomethylated transcripts across all brain subregions, as well as the region-specific hypomethylated transcripts. Dots and lines denote the displayed comparison and bars show the number of transcripts contained in the overlap. C. ACC - anterior cingulate cortex, DG - dentate gyrus, 5UTR - 5' untranslated region, 3UTR - 3' untranslated region, CDS - coding sequence, ncRNA - non-coding RNA.

Supplemental Figure 6

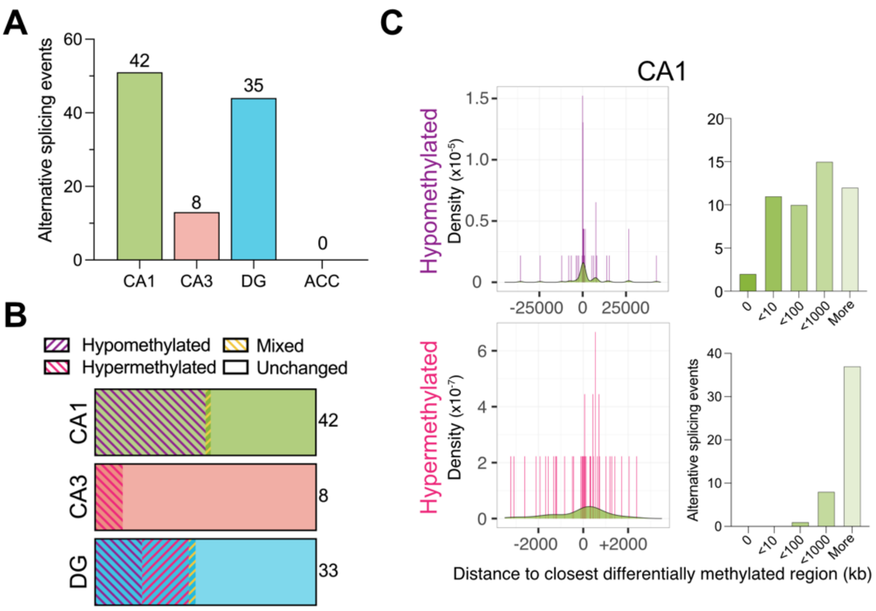

Figure S6. Differential splicing events cannot explain the m^{6}A hypomethylation detected in the aged mouse hippocampus. **A.** Bar graph showing alternative splicing events in the corresponding brain region when comparing old against young mice. The numbers on top of the columns refer to the number of affected transcripts. **B.** Bar plot showing the transcripts with alternative splicing events. The patterns show the fraction of these transcripts that are also hyper- or hypomethylated or are categorized as mixed transcripts, displaying the limited overlap between this populations. **C.** Distance to the closest differentially methylated region for all alternative splicing events in the CA1 in old vs. young mice, showing that splicing and methylation sites are too distant to influence each other. All distances are expressed in kilobases (kb).

Supplemental Figure 7

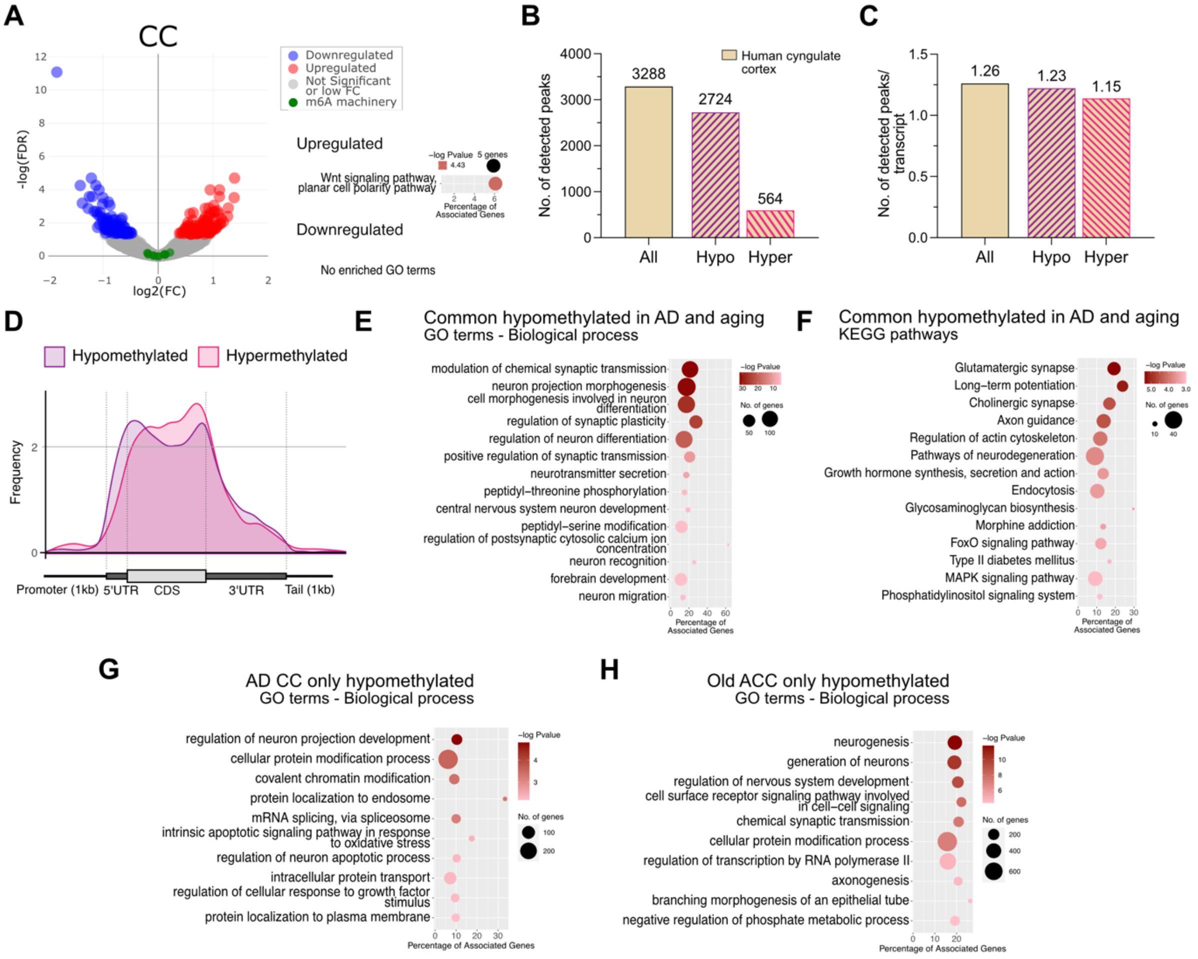

Figure S7. Altered m^{6}A RNA methylation in the cortex of human AD patients. A. Volcano plot displaying the changes in gene expression observed in the human CC comparing AD patients and control individuals (FC > 1.2, FDR ≤ 0.05). Highlighted in green are the known m^{6}A writers, readers and erasers, showing that their mRNA levels are not affected in our dataset. Right panel shows significantly enriched GO terms (Biological process) for upregulated genes. Downregulated genes resulted in no enriched GO terms. **B.** Total number of differentially methylated m^{6}A peaks in AD compared to control samples, as well as hypo- and hypermethylated peaks. **C.** Bar plot showing the average number of altered m^{6}A peaks per differentially methylated transcript when comparing AD to control. **D.** Guitar plot showing the distribution frequency of hypo- and hypermethylated m^{6}A peaks along mRNA transcripts. **E, F.** Enriched GO terms (Biological process, E) and enriched KEGG pathways (F) for m^{6}A hypomethylated transcripts detected in the CC of human AD patients and the ACC of aged mice (converted to their corresponding human homolog). **G.** Enriched GO terms (Biological process) for m^{6}A hypomethylated transcripts detected specifically in the human cortex of AD patient but not

in the ACC of aged mice. **H.** Enriched GO terms (Biological process) for m^{6}A hypomethylated transcripts detected specifically in the ACC of aged mice but not in the human cortex of AD patient. CC - cingulate cortex, 5'UTR - 5' untranslated region, 3'UTR - 3' untranslated region, CDS - coding sequence, ncRNA - non-coding RNA.

Supplemental Figure 8

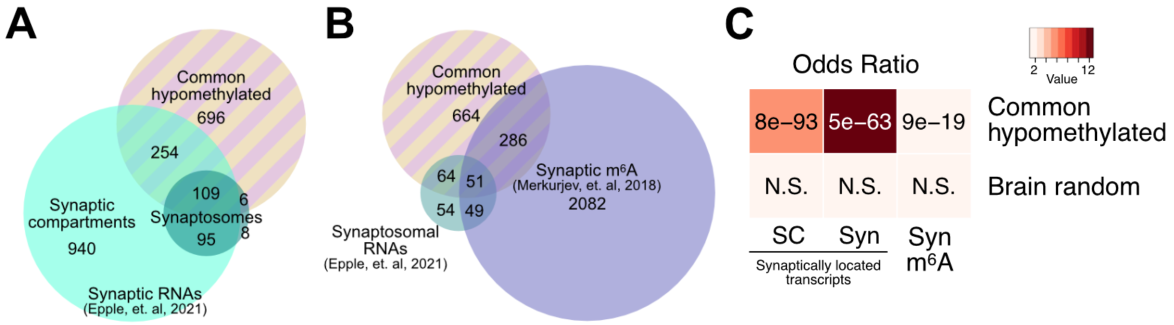

Figure S8. Transcripts displaying m^{6}A hypomethylation in the aging mouse and human AD brain are enriched at synapses. A. Venn diagram showing the overlap between commonly hypomethylated transcripts depicted in main Fig 4E and synaptic-localized RNAs detected in hippocampal synaptosomes or synaptodendritic compartments of neurons cultured in microfluidic chambers. **B.** Venn diagram comparing m^{6}A hypomethylated transcripts commonly detected in human AD patients and aged mice to synaptosomal RNAs from Epple, *et. al.* (2) as well as synaptic (syn) m^{6}A transcripts described by Merkurjev, *et. al.* (3). **C.** Heat map showing the enrichment (odds ratio) and significance of the overlaps displayed in A and B. Colors represents odds ratio of the comparison while the numbers refer to the p value. “Brain random” corresponds to a list of 2000 randomly selected brain-expressed transcripts, included as control. SC – synaptic compartments, Syn – synaptosomes, Syn m6A – synaptic m^{6}A mRNAs, N.S. – not significant.

Supplemental Figure 9

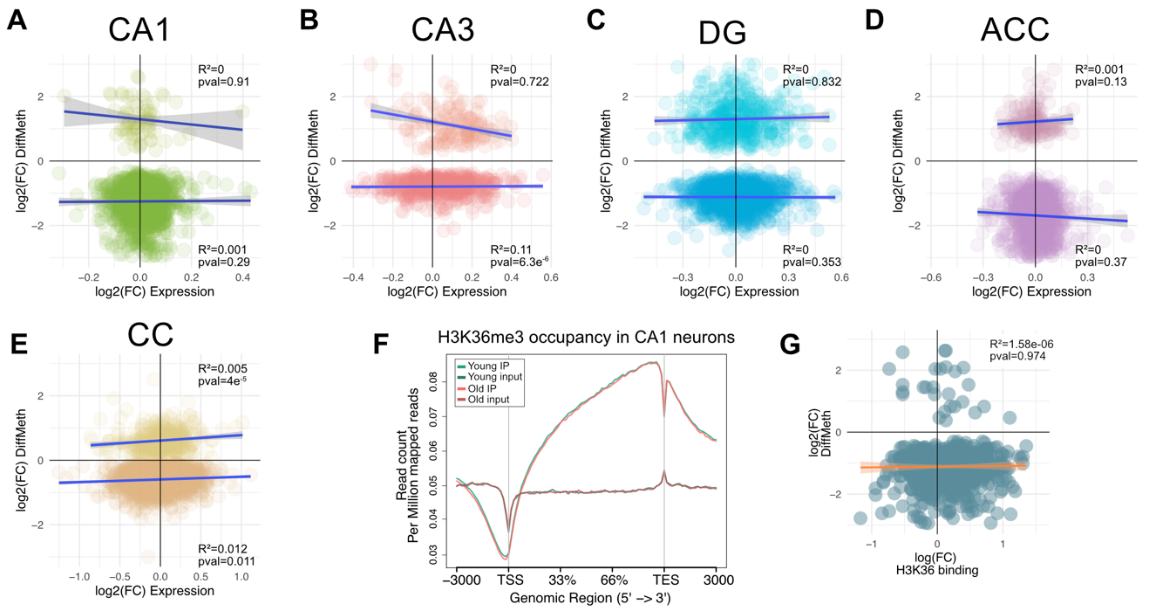

Figure S9. Changes in m^{6}A RNA-methylation are not linked to altered mRNA levels or H3K36me3. A-E. Scatter plots showing the correlation between observed changes in m^{6}A RNA-methylation and changes in the corresponding transcript levels in the CA1 (A), CA3 (B), DG (C), and ACC (D) regions of 3 vs 16 months old mice as well as in the human CC (E) of control vs AD patients. **F.** Occupancy of H3K36me3 in CA1 neuronal nuclei was determined by ChIP-seq. The curves show normalized reads mapped to the displayed genomic regions for immunoprecipitated (IP) and input samples obtained from young and old mice. No difference was detected amongst groups. **G.** Scatter plot displaying changes in H3K36me3 occupancy and m^{6}A changes for the differentially methylated transcripts in the aged CA1. No significant correlation was detected. In all scatter plots, the line represents the best model to fit the data points, CI is also displayed. Reads in CPM. TSS = transcription start site, TES = transcription end site.

Supplemental Figure 10

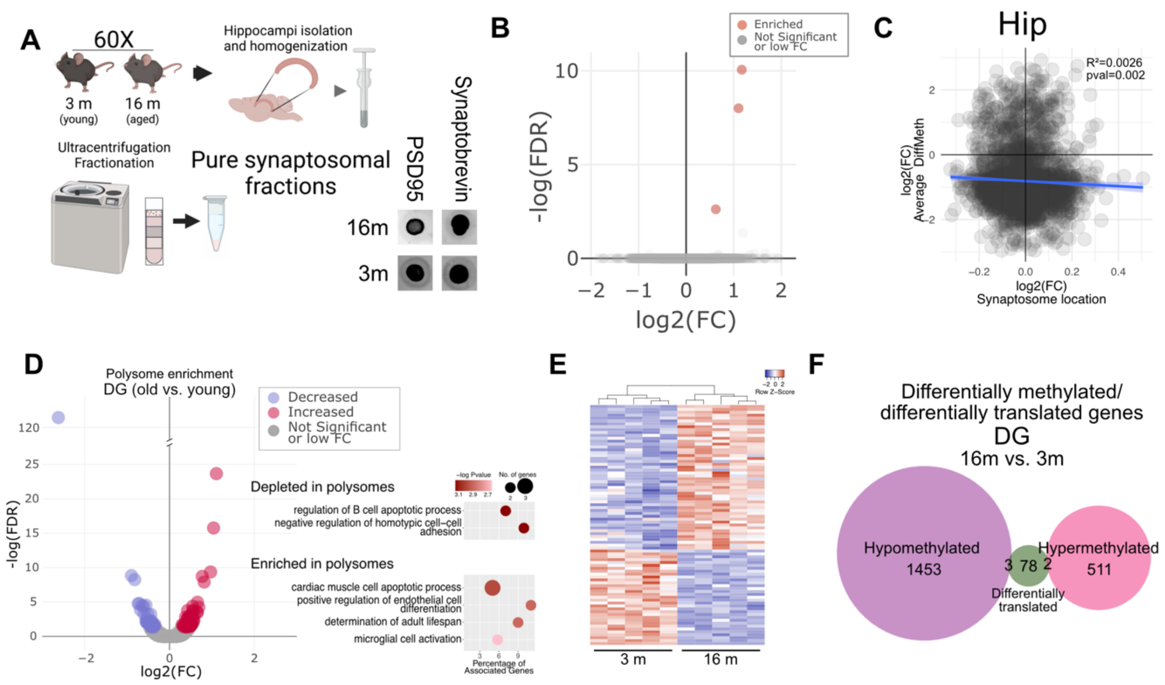

Figure S10. Synaptosomal and polysomal RNA compared in the young vs aged brain. A Diagram showing synaptosomal fraction purification from the hippocampus of young (3 month) and old mice (16 month) as described previously (2). Synaptosome purity was confirmed by dot blot using the synaptic markers PSD95 and synaptobrevin. **B.** Volcano plot showing differential expressed synaptosomal RNAs in the old vs. young hippocampus. Only three genes were significantly different. **C.** Scatter plot showing that there is no significant correlation between changes in the expression level of synaptosomal transcripts and changes in their methylation level. No FC cutoff but only FDR <0.05 cutoff was applied for differential methylation and differential expression. **D.** Volcano plot displaying the results of a differential expression analysis performed on polysomes isolated from the hippocampal DG region of young (3 month) and old mice (16 month). The right panel shows GO terms (Biological process) for mRNAs enriched or depleted from polysomal fractions in aged mice (right panel). **E.** Heatmap displaying the differentially translated mRNAs across the analyzed polysome samples. **F.** Venn diagram comparing differentially translated and differentially methylated mRNAs in the aged DG reveals little overlap between these populations.

Supplemental Figure 11

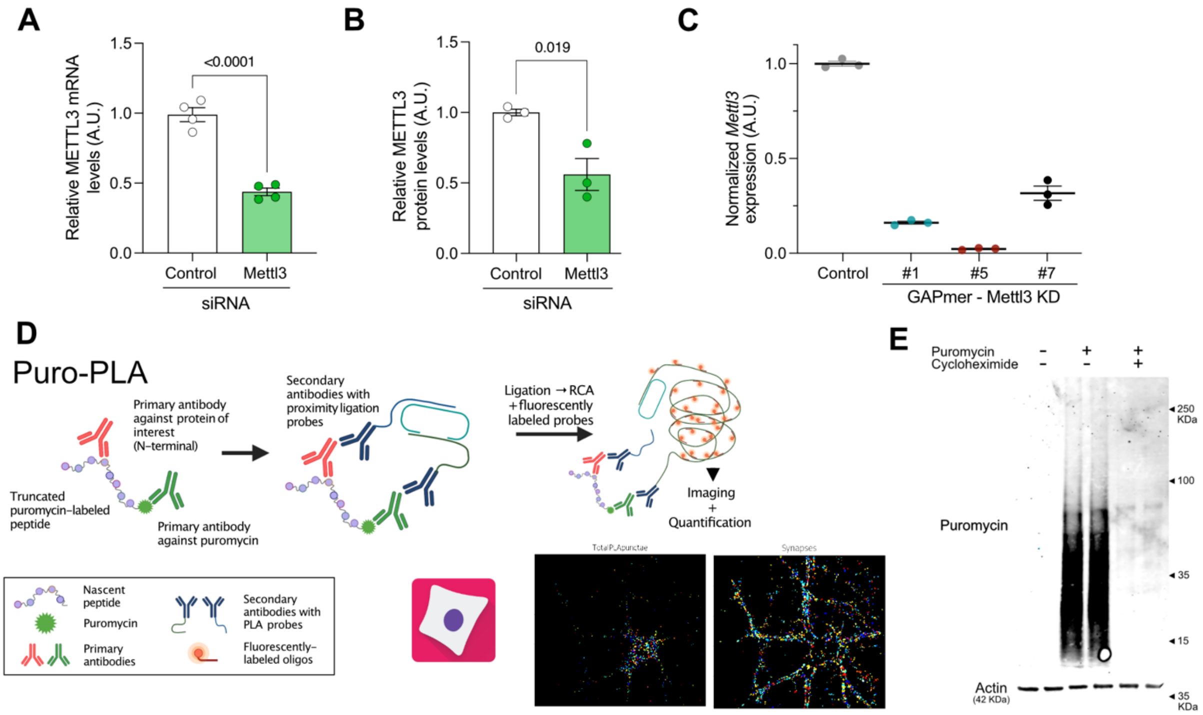

Figure S11. Knockdown of *Mettl3* via GAPmers to study local protein synthesis is superior to siRNA approaches A. Bar graph showing that *Mettl3* knockdown via siRNAs only partially decreases *Mettl3* mRNA at and protein levels **(B)** in hippocampal neurons when assayed 48 after treatment. **C.** Validation of LNA GAPmer-dependent knockdown of *Mettl3* in primary hippocampal neurons. Shown are the mRNA levels of Mettl3 after 48 hours treatment with control or Mettl3-targeting GAPmers (1,5 and 7). GAPmer #5 was selected for further experiments. Graphs in A-C display the mean +/- SEM of each condition. Each data point represents one independent replicate, statistical significance was determined by Student's t test. **D.** Scheme of the Puro-PLA process. Shown are representative images from the automated Puro-PLA and synapse detection pipeline using Cell Profiler. **E.** Validation of puromycin incorporation into nascent protein chains. Western blot using a puromycin antibody showing the labeling of proteins and the function of the cycloheximide pretreatment to cause translational arrest.

Supplemental Figure 12

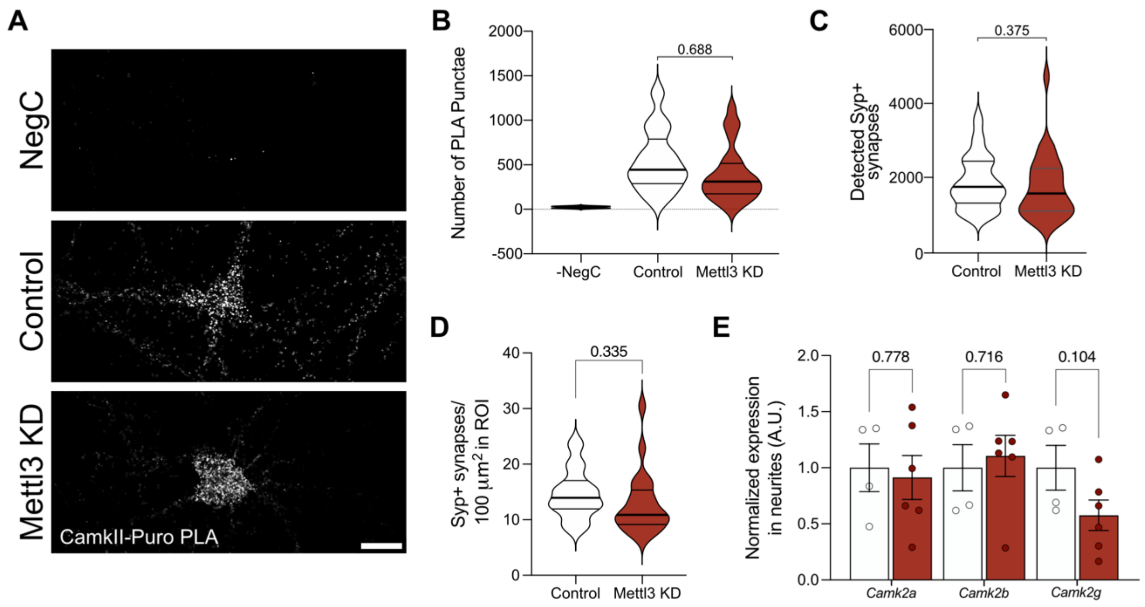

Figure S12. Mettl3 knockdown does not affect the bulk amount of CAMKII production, synapse number or synaptic levels of *CamKII* transcripts. A. Representative confocal images from untreated (without puromycin treatment, NegC), control and Mettl3 GAPmer-treated cells showing CAMKII-Puro PLA punctae. Scale bar: 10µm. **B.** Violin plots showing the total number of detected CAMKII-Puro PLA punctae detected in Puromycin-, control and *Mettl3* KD neurons. **C, D.** Total number of synapses (C) and number of synapses normalized for the analyzed area (D) was assayed via synaptophysin immunostaining (SYP+) in control and *Mettl3* KD neurons. Graphs in B, C and D show the mean of 3 independent experiments. For each experiment 7-13 neurons were imaged and analyzed. Individual data points were used to generate the violin plot. Quartiles are marked by gray lines. Statistical significance was determined by Student's t test on the mean values of each independent replicate. **E.** mRNA levels, as determined by qPCR, of different *CamKII* isoforms in the synaptic compartment of neurons grown in microfluidic chambers were analyzed after treatment with control or *Mettl3* GAPmers. Graphs display the mean +/- SEM of each condition. Each data point represents one independent replicate, statistical significance was determined by Student's t test

Supplemental Figure 13

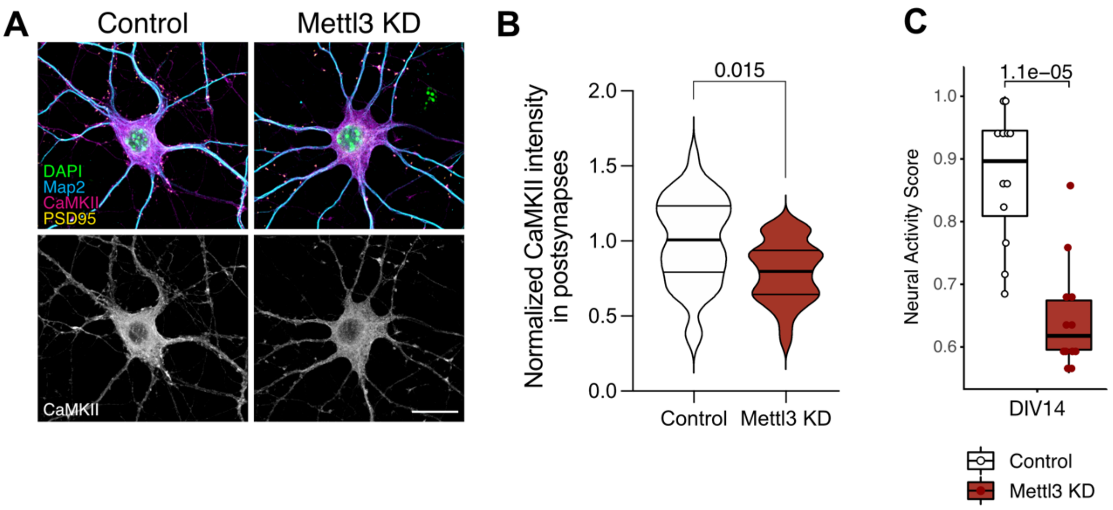

Figure S13. A. ***Mettl3*** **knockdown reduces synaptic CAMKII levels and impairs neuronal activity. A.** Representative images showing CAMKII immunohistochemical signal along with MAP2 and PSD95 to visualize synapses and dendrites in *Mettl3* KD neurons and control cells. Scale bar is 20 µm.**B.** Quantitative analysis of the CAMKII signal revealed reduced synaptic CAMKII levels in *Mettl3* KD neurons. n=4, 8-10 neurons per replicate. Significance determined by t test performed on the means of independent replicates. **C.** Bar plot showing the neural Activity Score obtained from MEA recordings of *Mettl3* KD and control treated primary neurons at DIV 14 (n=12, *t*-test).

Supplemental Figure 14

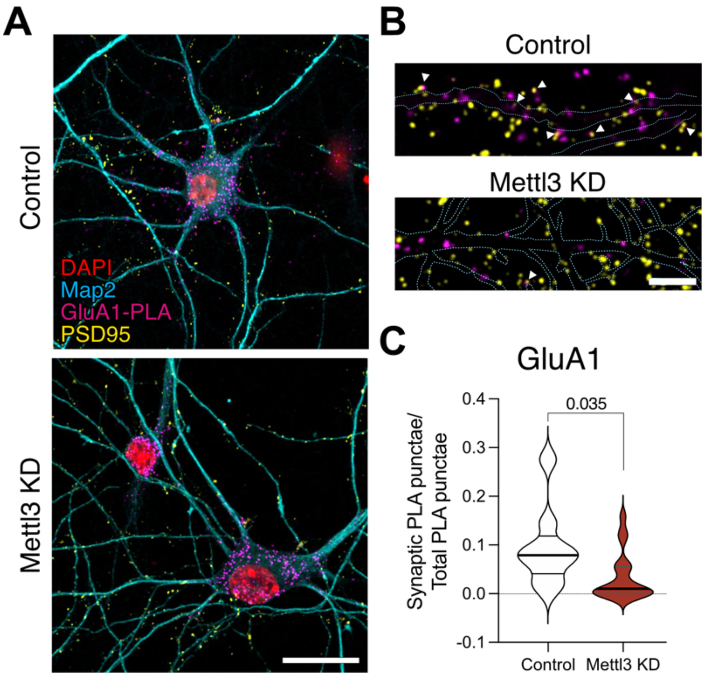

Figure S14. Mettl3 knockdown impairs the synaptic translation of *GluR1* mRNA. A. Representative images sowing primary neurons labeled by Puro-PLA for *GluA1*. DAPI is displayed in red, Map2 in cyan, GluA1-PLA in magenta and PSD95 in yellow. Scale bar is 20 µm. **B.** High magnification images of a representative GluA1-PLA assay. Highlighted by arrowheads is the location of PLA puncta (magenta) at postsynaptic compartments (PSD95+, yellow). Map2+ dendrites are outlined in cyan. Scalebar is 5 µm. **C.** Violin plot showing the quantification of synaptic-localized GluA1-PLA punctae in control (white) and *Mettl3* KD (dark red) treated primary neurons. n=3, 10 neurons analyzed per replicate. Significance determined by *t*-test performed on the means of independent replicates.

Supplementary methods

Animals

Three- (young) and 16- (old) mo-old male mice were purchased from Janvier Labs. Animals were housed under standard conditions. Experiments were performed according to the protocols approved by the Lower Saxony State Office for Consumer Protection and Food Safety.

Aged brain subregions dissection

The hippocampus of 10 young and 10 old mice was dissected into its corresponding subregions - CA1, CA3 and DG - using an 18G needle. The ACC was isolated with a scalpel. All dissections were performed on ice and the isolated tissue was flash-frozen in liquid nitrogen. Samples remained in -80˚C until right before RNA extraction.

RNA extraction

RNA used for sequencing was extracted from tissue using the NucleoSpin RNA/Protein Kit (Macherey-Nagel), according to the manufacturer's instructions.

For all other applications, cell or tissue samples were homogenized in an appropriate volume of TRI reagent (Sigma) using a Bead Ruptor Elite for 30 s with 0.5 mm ceramic beads. RNA was isolated using the Direct-zol RNA miniprep kit or the Clean and concentrator -5 (Zymo Research) according to the manufacturer's instructions. RNA was eluted in nuclease-free water in a volume of 6-50 µl.

RNA concentrations were determined by Nanodrop (Thermo) or Qubit with RNA HS Assay Kit (Thermo). For samples used for sequencing, RNA integrity was assayed by electropherogram in a Bioanalyzer using a total RNA Assay with a Pico/Nano Chip (Agilent). RNA samples were always kept on ice and stored at -80˚C when not in use to prevent degradation.

meRIP

Mouse RNA samples were processed as previously described for meRIP-seq (1). Briefly, isolated RNA was depleted of ribosomal RNA (rRNA) using the NEBNext ribosomal RNA removal kit (NEB), scaling up according to the initial amounts of RNA. The rRNA-depleted RNA from three or four mice was pooled together for each replicate, for a total of three replicates per condition and fragmented for 8 minutes at 70˚C to an average fragment size of ~80 nt using Fragmentation Reagents (Invitrogen).

After each step, the RNA was cleaned using the Clean and Concentrator kit and eluted in an appropriate level of nuclease-free water. rRNA depletion, fragment size and RNA quality were controlled in the Bioanalyzer, using Pico, Nano or Small RNA chips as needed.

10 µg of anti- m^6A antibody (Synaptic Systems) or rabbit IgG control (Millipore) were added to 50 µl of Protein A/G beads (Thermo) and incubated in 500 µl of IP buffer (0.2 M Tris-HCl pH 7.5, 0.5 M NaCl, Igepal 2%) for 4 hours at 4˚C with constant rotation. 10 µg of fragmented RNA per replicate were used for the subsequent RIP, with 500 ng (5%) kept to serve as the input. The RNA was incubated with the antibody-beads conjugate, in 1 ml of IP buffer supplemented with 200 units SUPERase-in (Invitrogen) overnight (ON) at 4˚C. Beads were washed 5 times with IP buffer and precipitated RNA was eluted with 6.7 mM m^6A in 200 µl IP buffer for 1 hour at 4˚C with agitation. Eluted RNA was cleaned before proceeding to library preparation.

Due to their low starting RNA concentration, human RNA samples were processed in a slightly different manner, based on a previously published protocol (4). 5-10 µg of purified total RNA were depleted from rRNA using the RiboMinus Eukaryote Ribosomal Removal Kit (Invitrogen) and fragmented for 5 minutes at 70˚C to a fragment size of 100-120 nt with Fragmentation Reagents. RNA was cleaned using the Clean and Concentrator Kit after every step and eluted in an appropriate volume of nuclease-free water. 500 ng of fragmented RNA were used for each IP, keeping 5% as input. 3 µg of anti- m^6A antibody was conjugated with 30 µl of Protein A/G beads for 2 hours at 4˚ C with rotation in 500 ul IP buffer. After washing the beads, RNA was added in 500 ul of IP buffer supplemented with 200 units SUPERase-in and incubated ON at 4˚C with rotation. Beads with immunoprecipitated RNA were washed 5 times with IP buffer and further washed with low-salt (50 mM Tris pH 7.4, 50 mM NaCl, 1mM EDTA, 1, 0.1% NP-40, 0.1% SDS) and high-salt (same as low-salt but with 500mM NaCl) buffers at 4˚C with rotation to remove nonspecific binding. RNA was eluted with 6.7 mM m^6A in 200 µl IP buffer for 1 hour at 4˚C with agitation. Eluted RNA was cleaned before proceeding to library preparation.

Library preparation and sequencing

Mice samples (including meRIP-seq, polysome sequencing and synaptosomal RNA-Seq) were prepared for sequencing using the TruSeq Stranded Total RNA Library Prep Kit (Illumina) according to the manufacturer's instructions. All of the RNA obtained from IP samples was used for library preparation, for input samples 300 ng was used. Libraries were amplified for a total of 13 cycles.

Human samples were prepared using the SMARTer Stranded Total RNA Kit v2 - Pico Input Mammalian (Takara) according to the manufacturer's instructions. Since samples were already fragmented, the fragmentation step was skipped. All of the RNA obtained from IP samples was used for library preparation, for input samples 2 ng were used. Libraries were amplified for 12 (input) or 16 (IP) cycles.

Prepared libraries were sequenced in a Hiseq 2000 System (Illumina) for 50 cycles in single-end reads.

qPCR

cDNA synthesized from highly abundant samples was prepared using the Transcriptor cDNA first strand Synthesis Kit (Roche) using 100 ng-1 µg of total RNA or 5-20 ng of rRNA-depleted RNA as starting material. The manufacturer's protocol for cDNA synthesis was followed, with a combination of random hexamers and Poly(dT) oligos.

immunoprecipitated samples and low abundance samples were synthesized using the Maxima first strand cDNA synthesis Kit (Thermo), according to the manufacturer's instructions. 1-10 ng of rRNA-depleted RNA or the full amount of IP RNA were used as input.

Synthesized cDNA was diluted 1:5-1:10 with nuclease-free water before being used for qPCR. Reactions were run in a Light Cycler 480 (Roche) in 96- or 384-well plates, using the Light Cycler 480 SYBR Master Mix (Roche). Each reaction was run in duplicate, in a volume of 20 µl and using 4 µl of cDNA per reaction. Primers used were custom designed, validated and used at a final concentration of 0.5 µM. Reactions were run for a maximum of 45 cycles with a reference gene in every plate and quantified as expression relative to the reference (and input in the case of IPs). 3-6 biological replicates were used in every case and statistical differences were determined by a t test, unless otherwise indicated.

Hippocampal primary neuronal culture

Pregnant (embryonic day 15: E15) CD1 mice were purchased from Janvier Labs and sacrificed under anesthesia by cervical dislocation on day E17. The pups were dissected and their hippocampi were isolated and collected in ice-cold DPBS (pH 7.4, without Ca and Mg). The hippocampi of 8-14 pups were used to prepare the primary cultures using a mild dissociation protocol with papain to prevent cell death and increase neuronal yield. The Papain Dissociation System (Worthington Biochemical) was used according to the manufacturer's instructions with an incubation time of 45 minutes. The single-cell suspension was counted using a hemocytometer and cells were plated at a density of 30-40,000 cells/cm^2 in Neurobasal Plus Medium (Gibco) supplemented with 1X B27 Plus Supplement (Gibco), 1X

PenStrep (Gibco) and 1X GlutaMAX (Gibco). Culture plates were kept at 37˚C and 100% humidity with 5% CO_2. Cells were supplemented with fresh medium after 5 days and half the medium was exchanged once a week after that.

siRNA/ LNA GAPmer transfection

Pre-designed control and Mettl3-targeting siRNAs were purchased from Origene, control and Mettl3-targeting LNA GAPmers were designed and purchased from Qiagen. 2 pmol of the corresponding control/Mettl3 siRNA/GAPmer were packaged into lipid nanoparticles (LNPs) specially formulated to deliver RNAi into primary mouse neurons using the Neuro9 siRNA Spark Kit (Precision Nanosystems). Cells were transfected with 0.3 µg/ml of siRNA/GAPmer supplemented with 1µg/ml ApoE4 on DIV 7. A fluorescent control siRNA was used to confirm a transfection efficiency of more than 80%. Knockdown efficiency was initially validated by qPCR after 48 hours but sufficient decrease in Mettl3 protein and m^6A levels were reached with the use of GAPmers for 6 days after transfection. Before fixation or RNA or protein extraction cells were washed with sterile DPBS to remove medium.

Western blot

Cells or tissue were dissociated in an appropriate volume of RIPA buffer (140 mM NaCl, 1 mM EDTA, 0.1% sodium deoxycolate, 1% Triton X-100, 10 mM Tris pH 8, 1% SDS) supplemented with 1X complete proteinase inhibitor (Roche). Protein concentration was quantified using the Pierce BCA Protein Assay Kit (Thermo) and 30 µg of protein were used per well of precast 4-15% polyacrylamide gels (Bio-Rad). Gels were run for 20 minutes at 90V followed by 50 minutes at 120V in Running buffer (25 mM Tris, 190 mM glycine, 0.1% SDS) and transferred into PVDF membranes (Bio-Rad) using the Trans-Blot Turbo Transfer System (Bio-Rad) in Auto mode.

Membranes were washed with PBST (1X PBS, 0.05% Tween-20) and blocked for 1 hour at room temperature (RT) in 5% BSA. Primary antibodies diluted in 5% BSA were incubated ON under shaking at 4˚C. Fluorescent secondary antibodies (IRDye, LI-COR) were incubated for 2 hours at RT with shaking. Blots were imaged by fluorescence with an Odyssey DLx (LI-COR) and the resulting images were quantified with ImageStudio.

m^6A quantification

m^6A concentration was determined using a m^6A Methylation Assay Kit Fluorometric (Abcam). The starting material was 200 ng of rRNA-depleted RNA and the manufacturer's protocol was followed. All reactions were carried out in duplicate and a standard curve of m^6A /A was included to have

quantitative results. Reactions were read in a FLUOstar Omega Multiplate reader (BMG) in fluorescence mode.

Immunofluorescence

Cells used for imaging were seeded on etched coverslips (2 hours in nitric acid, followed by 1 hour washing and 2 hours in 70% ethanol, kept in sterile water) coated with 0.5 mg/ml poly-D-lysine ON at 37˚C. Cells were fixated for 10 minutes in fixating solution (4% paraformaldehyde, 4% sucrose in 1X PBS).

Coverslips were permeabilized using T-PBS (0.3% Triton-X100 in 1X PBS) for 30 minutes at RT followed by 1 hour in blocking solution (5% BSA in T-PBS). Primary antibodies were incubated ON at 4˚C with shaking and secondary antibodies for 2 hours at RT. All antibodies were diluted in T-PBS.

Microscopy

Images were captured in a Leica dmi8 microscope fitted with a STEDycon STED/Confocal (Abberior). Immunofluorescence and Puro-PLA images were acquired in the confocal mode, with a 63X oil immersion objective and using identical acquisition settings for all images to be compared.

Polysome sequencing

Polysomes were prepared from the DG of five young and five old animals as described (17). Briefly, tissue samples were lysed in Polysome buffer (50 mM Tris-HCl pH 7.4, 10 mM $MgCl_2$, 100 mM NaCl, 1% Triton-X-100, 1 mM DTT) supplemented with RNase inhibitors and 100 µg/ml cycloheximide, using a MICCRA D-1 homogenizer. Cell debris was removed by centrifugation at 20,000 x g for 10 min at 4°C. The soluble whole cell extracts were separated on 10-50% sucrose density gradients (prepared in Polysome buffer with 100 µg/ml cycloheximide) in an SW40Ti rotor by centrifugation for 3 h at 35,000 rpm. Fractions containing polysomes (determined by monitoring the absorbance of each fraction at 260 nm) were pooled and RNAs were extracted using phenol:chloroform:isoamyl alcohol (25:24:1), ethanol precipitated and resuspended in water.

Synaptosome isolation for sequencing

Synaptosomes were isolated from the hippocampi of 3- and 16-months old mice as recently described (2).

H3K36me3 ChIP

Cell type specific chromatin isolation and ChIP sequencing was performed as previously described (18, 19). 3-4 CA1 were pooled for each replicate and nuclei were FACS sorted by NeuN expression. 300 ng of chromatin and 1 µg of H3K36me3 antibody (Abcam, ab9050) were used for each ChIP. Libraries were prepared with the NEBNext Ultra II DNA library preparation kit, 8 cycles of PCR amplifications were performed for both inputs and IP samples. Average fragment size of the libraries was 380 bp.

Puro-PLA

The PLA was performed using the Duolink proximity ligation assay kit red (Merck) according to the manufacturer's instructions. Primary antibodies against puromycin and the protein of interest (targeting the N-terminal region) raised in different species were used for the PLA and incubated ON at 4 °C. Counterstain antibodies (Map2 and SYP) were incubated ON at 4 °C along with the PLA primary antibodies, and an additional incubation step was added after finishing the puro-PLA protocol to add the secondary antibodies for the counterstains for 2 h at RT before mounting.

For each condition and replicate, 7 to 13 neurons were captured by confocal imaging and analyzed using Cell Profiler to automate the analysis and remove biases. Background-level PLA signal was adjusted to samples without puromycin treatment.

Microfluidic chambers for synaptic RNAs

Custom made polydimethylsiloxane (PDMS) microfluidic chambers optimized for the harvesting of synaptic RNAs were produced as previously published (2). 70,000 cells were seeded on every side of the chamber and cultured for 7 days with daily medium exchange between the distinct compartments and replenishment to counteract evaporation. On DIV 7 the somas of cultured neurons on both the dendritic and axonal side of the chambers were treated with LNPs containing Control or Mettl3 KD LNA GAPmers. Cells were cultured for a further 6 days with constant medium replenishment before harvesting the synaptic RNAs. The synaptic compartment was excised using a purpose-built device and RNA was purified using the GenElute Total RNA Purification Kit (Sigma).

Synapse quantification

Primary neurons were infected with an AAV8-CamKII-mCherry virus on DIV3 (UNC Vector Core) at a final concentration of $3.375*10^{12}$ viral particles per 100,000 cells. Treated cells were then transfected with LNPs containing the Mettl3 KD or Control LNA GAPmers on DIV7, as described previously. Cells were fixed on DIV12 and stained with a VGLUT1 antibody and a PSD95 nanobody. Stained neurons were imaged by confocal microscopy and the colocalization of VGLUT1+/PSD95+ regions quantified

using a custom automated pipeline in Cell Profiler, filtered to the mCherry signal and normalized to the dendrite length.

Multielectrode arrays

Multielectrode array (MEA) recordings were performed in the Maestro system (Axion) on 24-well plates. 150,000 cells were seeded in 6 µl on plates coated overnight at 37˚C with 0.1% PEI and supplemented with 1 µg/ml Laminin. After 1 hour at 37˚C and 5% CO_2, 500 µl Neurobasal plus medium was added. Cells were transfected with LNPs containing the Mettl3 KD or Control LNA GAPmers on DIV7, as described previously. Recordings were performed once a day until DIV14, after 10 minutes of acclimation for 5 minutes with the following settings: band-pass filter (Butterworth, 300-500 Hz), spike detector (adaptive threshold crossing, 8xSD), burst detector (100 ms max inter-spike interval, 5 spikes minimum, 10 spikes min for network bursts, 10 ms mean firing rate detection window), statistics compiler. Recordings were exported using Axion's Neural Metric Tool and the resulting files were processed with a custom pipeline to calculate the corresponding Neural Activity Score as described previously (20).

Supplemental Literature

1. T. Berulava *et al.*, Changes in m6A RNA methylation contribute to heart failure progression by modulating translation. *European Journal of Heart Failure* **22**, 54-66 (2020).
2. R. Epple *et al.*, The Coding and Small Non-coding Hippocampal Synaptic RNAome. *Mol Neurobiol.* **58**, 2940-2953 (2021).
3. D. Merkurjev *et al.*, Synaptic N6-methyladenosine (m6A) epitranscriptome reveals functional partitioning of localized transcripts. *Nature Neuroscience* **21**, 1004-1014 (2018).
4. Y. Zeng *et al.*, Refined RIP-seq protocol for epitranscriptome analysis with low input materials. *PLOS Biology* **16**, e2006092 (2018).
5. R. C. Team, R: A language and environment for statistical computing. (2013).
6. S. Heinz *et al.*, Simple combinations of lineage-determining transcription factors prime cis-regulatory elements required for macrophage and B cell identities. *Mol Cell* **38**, 576-589 (2010).
7. R. G. Cavalcante, M. A. Sartor, annotatr: genomic regions in context. *Bioinformatics (Oxford, England)* **33**, 2381-2383 (2017).
8. X. Cui *et al.*, Guitar: An R/Bioconductor Package for Gene Annotation Guided Transcriptomic Analysis of RNA-Related Genomic Features. *BioMed Research International* **2016**, 8367534 (2016).
9. C. Sievert, *Interactive web-based data visualization with R, plotly, and shiny* (2019).
10. T. Hulsen, J. de Vlieg, W. Alkema, BioVenn – a web application for the comparison and visualization of biological lists using area-proportional Venn diagrams. *BMC Genomics* **9**, 488 (2008).

11. A. Khan, A. Mathelier, Intervene: a tool for intersection and visualization of multiple gene or genomic region sets. *BMC Bioinformatics* **18**, 287 (2017).
12. L. Shen, GeneOverlap: An R package to test and visualize gene overlaps. 10 (2019).
13. M. Kanehisa, Y. Sato, M. Kawashima, KEGG mapping tools for uncovering hidden features in biological data. *Protein Science: A Publication of the Protein Society* (2021).
14. J. Schindelin *et al.*, Fiji: an open-source platform for biological-image analysis. *Nature Methods* **9**, 676-682 (2012).
15. T. R. Jones *et al.*, CellProfiler Analyst: data exploration and analysis software for complex image-based screens. *BMC bioinformatics* **9**, 482 (2008).
16. L. Shen, N. Shao, X. Liu, E. Nestler, ngs.plot: Quick mining and visualization of next-generation sequencing data by integrating genomic databases. *BMC Genomics* **15**, 284 (2014).
17. H. Chassé, S. Boulben, V. Costache, P. Cormier, J. Morales, Analysis of translation using polysome profiling. *Nucleic Acids Research* **45**, e15 (2017).
18. A. Michurina *et al.*, Postnatal expression of the lysine methyltransferase SETD1B is essential for learning and the regulation of neuron-enriched genes. *The EMBO journal* **41**, e106459 (2022).
19. R. Halder *et al.*, DNA methylation changes in plasticity genes accompany the formation and maintenance of memory. *Nature Neuroscience* **19**, 102-110 (2016).
20. A. P. Passaro, O. Aydin, M. T. A. Saif, S. L. Stice, Development of an objective index, neural activity score (NAS), reveals neural network ontogeny and treatment effects on microelectrode arrays. *Sci Rep.* **11**, 9110 (2021).
